# Neighborhood Greenspace, Extreme Heat Exposure, and Sleep Quality over Time among a Nationally Representative Sample of American Children

**DOI:** 10.3390/ijerph21101270

**Published:** 2024-09-25

**Authors:** Rouzbeh Rahai, Nancy M. Wells, Gary W. Evans

**Affiliations:** 1 Human Centered Design Department, College of Human Ecology, Cornell University, Ithaca, NY 14850, USA; 2 Psychology Department, College of Human Ecology, Cornell University, Ithaca, NY 14850, USA

**Keywords:** child sleep quality, neighborhood greenspace, extreme heat exposure, heat mitigation, global climate change

## Abstract

Children’s sleep is essential for healthy development, yet over a third of children in the United States experience inadequate sleep. Environmental factors can influence sleep: greenspace exposure can promote better sleep, while heat exposure can disrupt sleep. As global climate change raises nighttime and daytime temperatures, greenspace may mitigate the negative effects of heat stress on sleep. We examined the direct effects of neighborhood greenspace and extreme heat exposure on sleep and the statistical interaction between greenspace and heat exposure on sleep outcomes among a nationally representative, four-year longitudinal sample of 8580 U.S. children ages 9–10 years at baseline. Hierarchical linear models incorporated a neighborhood greenspace measure: percent open park space within individual child census tracts, a measure of extreme neighborhood heat exposure during the summer months, and extensive individual and neighborhood-level covariates to test main and interaction effects on child sleep quality. Neighborhood open park space was related to better sleep quality, after controlling for covariates. Additionally, neighborhood extreme heat exposure was associated with worse sleep quality. A two-way interaction was found between neighborhood open park space and neighborhood heat exposure on sleep quality, suggesting open park space mitigated the negative effects of heat on sleep. The results indicate the potential contribution of open greenspace to improve child sleep and enhance resilience to extreme heat, which is an adverse outcome of climate change.

## 1. Introduction

**Figure 1:**
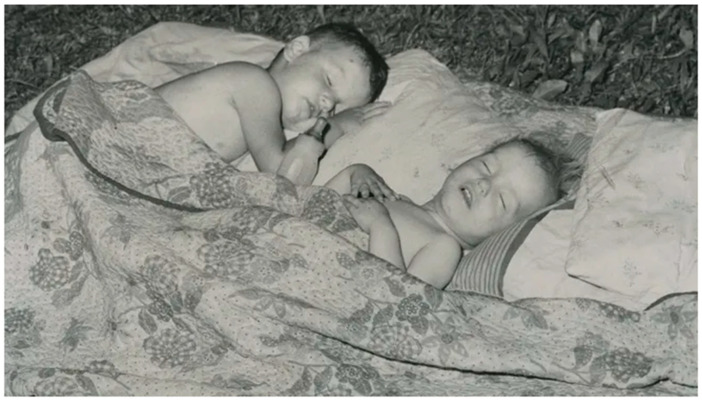
Children sleeping in Belle Isle Park, Detroit, Michigan under the stars in 1957 after their parents decided it was too hot to sleep at home [10]. Here, the natural environment is shown to be a place of refuge, providing comfort and relief when heat directly impacted children’s sleep. Nature can be a versatile space providing opportunities for adaptation to heat.

Children’s sleep is essential for healthy development, yet over a third of children in the United States experience inadequate sleep [1,2]. Adequate sleep is associated with improved cognitive functioning, emotion regulation, and both physical and mental health among children; whereas insufficient sleep increases risk of accidental injury, hypertension, obesity, and mental health and behavioral problems [3,4]. Insufficient sleep is differentially reported across the continental United States for both adults and children [2,5,6]. While child sleep is influenced by genetic factors and volitional behaviors of parents and children, a large proportion of inter-child sleep variability is explained by environmental factors [4,7]. Two important environmental factors found to influence sleep include greenspace exposure, which promotes better sleep [8], and ambient heat exposure, which disrupts sleep [9]. For instance, during periods of extreme heat, families have historically sought relief in natural environments, as depicted in Figure 1 where children slept outdoors in Belle Isle Park, Michigan, USA to escape the heat at home. In the present paper we examine whether greenspace can partially offset the adverse impacts of extreme heat on sleep quality among children.

Greenspace constitutes land area containing various forms of vegetation. Greenspace has been linked with a variety of health and wellbeing benefits among children [11,12] and is assumed to improve sleep by facilitating better mental and physiological health [13,14]. Greater proximate greenspace is positively associated with adult sleep quality; however, there are relatively few studies among children [9,15]. Research on children, greenspace, and sleep has yielded mixed results. A multi-year, cross-sectional study found that children ages 6–17 who reported having no access to parks and playgrounds in their neighborhoods had greater sleep problems than those with access to parks and playgrounds, after adjusting for demographic and household covariates [7]. A cross-sectional study of children living in Shanghai, China found that greater residential vegetation was significantly associated with earlier bedtimes on days without morning obligations, suggesting improved sleep patterns [15]. A study of Australian and German children found greater residential vegetation was associated with a lower prevalence of insufficient sleep among 10-year-old German children with various statistical controls, but the findings were not replicated in the rest of their sample of different ages [16]. Furthermore, some studies find positive associations between residential vegetation indexes and child sleep sufficiency using univariate models, but the results become nonsignificant in multivariate, adjusted models [14,16,17]. Other studies have found no direct effects of greenspace on sleep, but found that the association between greenspace and sleep differed by racial and socioeconomic subgroups [14,18,19].

A comprehensive review of heat and sleep finds that ambient indoor and outdoor heat significantly impacts sleep, especially during the hottest days and months worldwide [9]. Extreme heat events significantly affect indoor thermal comfort in residential settings, resulting in hot sleep environments [20] that can alter human core body temperature outside of the normal range, producing physiological responses that disrupt natural sleep–wake cycles and increase wakefulness [21,22]. The largest temperature-based sleep study to date examined 10 billion accelerometry-based sleep observations among adults across 68 countries and found that increases in nighttime temperature disrupt human sleep [23]. Much less research has explicitly explored temperature–sleep relationships among children, though studies suggest that children are more vulnerable to extreme heat than adults [10,24,25]. A UK study found that, during heatwaves, infants had multiple nighttime sleep problems compared to on non-heat wave nights [26]. A cross-sectional study of adolescents living in the U.S. documented higher temperatures associated with a decrease in nightly sleep efficiency, measured using accelerometry, after controlling for covariates [27]. Heat and sleep studies among children are nascent. 

The prevalence of excessive heat during sleep is rapidly escalating as people are experiencing warming temperatures and longer-lasting heat waves across all regions of the United States because of Global Climate Change [28,29]. Greenspace in neighborhoods has the potential to buffer the negative effects of heat on sleep. Vegetation provides significant cooling benefits through shading and evapotranspiration. Ground-level greening can reduce peak surface temperatures by 2–9 °C [30]. Greenspaces in cities have been shown to regulate air temperatures and, in some cases, lower nighttime temperatures by up to 4 °C [31]. Thus, greenspace can help produce more conducive nighttime sleep environments. 

### The Present Study

This study examines the direct associations between neighborhood greenspace and sleep, extreme heat and sleep, and the potential role of greenspace as a moderator of the effect of heat on sleep among a nationally representative sample of American children. To the best of our knowledge, no research has examined the interaction of greenspace and heat on sleep. In addition, we extend previous research by using a nationwide sample of individual children and by examining sleep measures over multiple years. Previous child-level research on greenspace, heat, and sleep is mostly cross-sectional [7,15]. We measure greenspace exposure at the census tract level, which is a more localized exposure measure than the relatively large buffer zone estimates used in previous research. 

Herein, we ask three primary research questions: Q1: Is neighborhood greenspace negatively associated with children’s sleep disturbances? Q2: Is extreme heat exposure positively associated with children’s sleep disturbances? Q3: Does neighborhood greenspace attenuate the significant negative effects of extreme heat on sleep? 

## 2. Method 

### 2.1. Participants

This research used data from the Adolescent Brain Cognitive Development longitudinal study (ABCD NDA 5.0 data release—https://nda.nih.gov/study.html?id=2313 (accessed on 10 September 2023; [32]), which recruited a representative sample of 11,878 children ages 9 and 10 years and participating caregivers once per year beginning in 2016 from 22 sample sites across the United States with comprehensive neurocognitive assessments [33,34]. In addition to collecting developmental data, the ABCD study has linked extensive, state-of-the-art environmental and community-level data using geospatial methods [35]. The 5.0 data release allowed linking access to various neighborhood characteristics including park area and heat. 

### 2.2. Sample Selection 

Our final analytical sample included 8580 children (72% of the original ABCD cohort) and 33,010 total data points from 5 testing years (baseline, 1-year follow up, 2-year follow up, 3-year follow up, and 4-year follow up) and across 22 sites. The 22 ABCD study sites are in primarily urban and suburban areas that span the Northeast, Midwest, South, and West regions of the United States. Given our focus on sleep outcomes, we began linking variables using the sleep database as the root database, which included 11,868 unique children. Participants were dropped from analysis due to absence of sleep, family income, parent education, and occupant data. In addition, participants were dropped if caregivers reported less than 100% of time spent at the primary residential address, to reduce exposure variance. A flow-chart of our sample reduction steps can be found in Supplemental File S1. 

### 2.3. Independent and Moderating Variables: Greenspace and Heat Measures

Independent and moderating variables included census tract level measurements of open park space and excessive heat based on children’s primary residential addresses. Open park space was defined as the percentage of public parkland within census tracts (total park area/census tract area) [36]. Data were collected by the National Neighborhood Data Archive (NaNDA) in 2018 where unique parks with open public access were identified that fully or partially overlapped with census tracts (NaNDA 2018). Excessive heat was measured as the number of days with a maximum temperature above 32.2 °C (90 °F) at the home address during the summer months [36,37]. Measuring excessive heat events during the summer months, as opposed to measuring temperature gradient, can capture acute impacts on the residential sleep environment and human thermoregulation, which can impact mental health and wellbeing [20,22,38]. 

### 2.4. Dependent Variable: Sleep 

Sleep was operationalized using total sleep disturbances from the parent-reported Sleep Disturbance Scale for Children (SDSC). The SDSC is a tool for evaluating sleep problems among school-aged children in clinical and non-clinical populations [39]. The SDSC is reliable (α = 0.71 to 0.79), and calculates total sleep problems by summing 26 parent reported items across 6 subscales, including scales of problems initiating and maintaining sleep, sleep breathing disorders, arousal and nightmares, sleep–wake transitions, excessive somnolence, and sleep hyperhidrosis [39]. The total score is the sum of the 26 items retained, with a possible range from 26 to 130 [39]. A review of subjective sleep measures in children concluded that this scale has good diagnostic accuracy based upon its psychometric properties, ease of scoring and access, and use by multiple investigators [40].

### 2.5. Covariates: Demographic and Neighborhood Factors

Child demographic covariates included age in months at the time of the testing year, sex, race/ethnicity, parent education level, and income-to-needs. Parent education level is based on the highest level from kindergarten to doctorate degree. Income-to-needs was not explicitly available in the ABCD data. We calculated income-to-needs by dividing the median value of each child’s combined family income band by the 2017 federal poverty line for the respective household size (income-to-needs ratio = household income / federal poverty threshold for household size. *Notes*: Household income was derived using the median of family reported income bands based on annual parent questionnaires on, “How much did you earn, before taxes and other deductions, during the past 12 months?”, with response choices, “1 = Less than $5000; 2 = $5000 through $11,999; 3 = $12,000 through $15,999; 4 = $16,000 through $24,999; 5 = $25,000 through $34,999; 6 = $35,000 through $49,999; 7 = $50,000 through $74,999; 8 = $75,000 through $99,999; 9 = $100,000 through $199,999; 10 = $200,000 and greater. 777 = Refuse to answer; 999 = Don’t know”. Household size was determined using annual parent questionnaires on, “How many people are living at your address? INCLUDE everyone who is living or staying at your address for more than 2 months”. Federal poverty thresholds using 2017 estimates were assigned based on the number of people in the house: $12,140 for a household of 1, $16,460 for 2 people, $20,780 for 3 people, $25,100 for 4 people, $29,420 for 5 people, $33,740 for 6 people, $38,060 for 7 people, and $42,380 for households of 8 or more people). Neighborhood level covariates included number of years living at primary residential address before baseline, census tract classifications of urbanicity (urban/urban clusters/rural), population density (persons per square mile), and neighborhood disadvantage. Neighborhood disadvantage is based on a composite index of census tracts’ socioeconomic disadvantage using income, education, employment, and housing quality data from the American Community Survey from 2013–2017 [35].

### 2.6. Statistical Analysis

We ran linear mixed models as our multi-level model using the lme4 package in R (Bates 2010). The equation underlying our general model can be found in Equation (1). By incorporating interaction terms including greenspace measures and heat exposure in our analysis, these variables statistically serve as covariates in our model at the child level. We controlled for clustering effects associated with each child, event year, and testing site to account for repetitive child measures and the nested structure of the data.

Total Sleep Disturbances ~ Intercept + Parent Education Level + Race/Ethnicity + Sex + Age + Income-to- needs + Years at Primary Address + Neighborhood Population Density + Neighborhood Disadvantage + Urbanicity + Neighborhood Open Park Space + Extreme Heat Exposure + (Neighborhood Open Park Space x Extreme Heat Exposure) + (1|Subject ID) + (1|Event Year) + (1|Site ID)(1)

We conducted a Variance Inflation Factor (VIF) test for multicollinearity and ensured VIF’s < 2 [41]. All continuous variables were mean-centered and scaled for comparable interpretation with the dependent variable. Our model used complete case analysis. Sensitivity analysis confirmed our current model outperformed the IQR-transformed model in terms of key metrics, including AIC, RMSE, and R^2^ (Supplemental File S2). All data preparation, processing, and results generation was done using R version 4.3.1 and is made publicly available in such a way that anyone with ABCD data access can reproduce the results (Supplemental File S2). 

## 3. Results

### 3.1. Child and Neighborhood Descriptive Characteristics

We analyzed data on 8580 unique children across 22 sites. Table 1 and Table 2 show descriptive statistics of categorical and continuous variables used in our analysis, respectively. In our sample, 48% were born as female. The mean age across all testing years was 140 months, with the minimum being 107 months and maximum 189 months; 54% reported identifying as White, 13% as Black, 20% as Hispanic, 2% as Asian, and 11% as Other; children were sampled from over 5 testing years, and 33,010 total observations were made, where the breakdown of observations across years were as follows: baseline (24%), 1-year follow up (23%), 2-year follow up (22%), 3-year follow up (21%), and 4-year follow up (10%) (Table 1). On average, caregivers reported a total sleep disturbance score of 36.3, with the minimum at 26 and maximum at 126. Children primarily lived in urban settings (87% Urban) (Table 1), and in households with average combined family incomes of 4.3 times the federal poverty level, with the minimum at 0.06 and the maximum at 21. On average, park area occupied 5% of children’s census tracts, with the minimum at 0% and maximum at nearly 77% (Table 2).

### 3.2. MLM Results: Greenspace & Extreme Heat Main Effects on Sleep

Our observed focal effects are organized under our primary research questions below. 

Q1: Is neighborhood greenspace negatively associated with children’s sleep disturbances?

Neighborhood proportional park area was negatively associated with children’s sleep disturbances (β = −0.02, SE = 0.01, *p* < 0.05), after controlling for covariates (Table 3). 

Q2: Is extreme heat exposure positively associated with children’s sleep disturbances?

Extreme heat was positively related to children’s sleep disturbances (β = 0.05, SE = 0.01, *p* < 0.01), after controlling for covariates (Table 3). 

### 3.3. MLM Results: Greenspace & Extreme Heat Interaction Effects on Sleep

Does neighborhood greenspace moderate the significant negative effects of extreme heat on sleep? 

**Figure 2:**
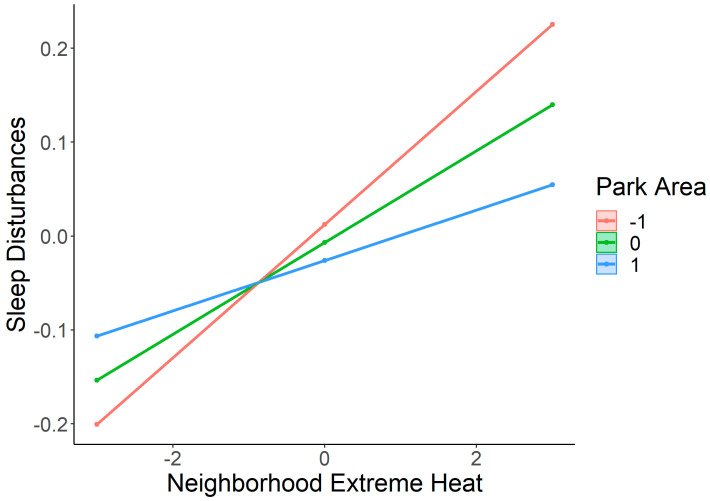
Interaction between neighborhood park space and extreme heat on sleep disturbances. The *X*-axis shows standardized extreme heat exposure (−3 to +3 SD), and the *Y*-axis represents sleep disturbances. Moderation lines indicate park space at −1, 0, and +1 SD. All values were mean centered and z scored for comparable interpretation.

As can be seen in Figure 2, the harmful relationship between extreme heat and sleep disturbances among children is mitigated by proximate open park areas (β = −0.02, SE = 0.01, *p* < 0.05), reflecting an interaction effect where greater park area reduces the impact of extreme heat, after controlling for covariates (Table 3).

## 4. Discussion

We found that neighborhood open park space was significantly associated with fewer sleep disturbances among children across multiple years, after controlling for individual and neighborhood factors that can influence sleep. (We found that a different greenspace measure, NDVI, was also significantly negatively associated with sleep disturbances, after implementing covariates. We did not, however, find a significant interaction effect between NDVI and extreme heat on sleep. We did not include results since NDVI measures include private, inaccessible areas of greenspace and NDVI ranges beyond the neighborhood boundary). Moreover, neighborhood extreme heat exposure during the summer months was significantly associated with greater sleep disturbances across multiple years. Furthermore, children living in neighborhoods with greater proportional open greenspace experienced fewer adverse impacts of extreme heat on sleep. On average, children living in neighborhoods with more proportional open park space experienced less sleep disturbances from extreme heat compared to those living in neighborhoods with less open park space (Figure 2). 

The potential practical implications of these findings can be discerned from the fact that a one standard deviation (SD) increase in the proportion of park area in children’s neighborhoods (about 8.8% coverage) was associated with a 0.02 SD decrease in sleep disturbances, translating to a 0.16-point reduction in total sleep disturbance scores. Similarly, a one SD increase in extreme heat exposure (about 26 days) was linked to a 0.05 SD increase in sleep disturbances, equivalent to a 0.39-point rise. Importantly, the moderation effect revealed that each additional SD of park area (8.8%) reduced the adverse impact of extreme heat by 0.02 SDs. This means that for children in neighborhoods with more greenspace, the harmful effect of extreme heat on sleep disturbances was attenuated by approximately 0.16 points for every SD increase in park area, highlighting a clear protective benefit of greenspace against heat-related sleep issues. While sleep disturbance is on a composite scale and challenging to interpret in day-to-day terms, even small shifts in the total score translate into substantial impacts on nightly sleep quality for children.

Our greenspace main effect findings are consistent with previous evidence linking neighborhood park access with fewer self-reported sleep problems [7] and greater neighborhood vegetation with improved sleep patterns among children [15,16]. Several studies have reported positive associations between spatial greenspace measures around the home and sleep quality and quantity among adults [9]. Few studies have utilized repeated measures to examine children’s sleep patterns over time. We use repeated measures in our analysis to understand environmental associations with sleep disturbances across different time points. Moreover, no prior studies have examined, as herein, a range of environmental and sleep measures among a national sample of American children. Previous null findings between greenspace and children’s sleep may be due to limitations in geographic sample or sleep measurement characteristics. For example, when children’s sleep is measured by self-report or parent report, few questionnaire items were used [7,15,16]. The current study uses a validated sleep scale of 27 items representing 6 subscale dimensions of sleep [39]. 

Our primary contribution demonstrates a significant interaction effect between proportional open park space in the neighborhood and heat on sleep (Both multiplicative interaction and additive models were tested. The multiplicative model explained more variance and showed a statistically significant interaction effect, while the additive model did not. More importantly, the multiplicative interaction test better fits our theoretical framework, capturing the context-dependent relationship where greenspace impact on sleep quality varies with heat exposure). Our interaction findings indicate that park space buffers the negative effects of heat on sleep. Public parks typically incorporate pervious surfaces, shade from trees, and water features that enhance their cooling effects. By providing open space, parks frequently augment air flow which also has a physical cooling effect. Public parks also provide opportunities for social interactions which can mitigate the negative impact of heat on sleep [42]. The social and psychological benefits of public parks, including reduced stress and increased physical activity, can further contribute to better sleep outcomes during periods of extreme heat [43]. Conceptually, greenspace can enhance human sleep by mitigating the negative environmental sequelae associated with extreme heat exposure [44]. Nature reduces environmental risk and bolsters human adaptation—how well a person responds in the face of adversity, resulting in positive developmental outcomes for children [44]. 

### 4.1. Limitations and Future Research 

Our study is not without its limitations. We measured available greenspaces around the home but were not able to measure actual use of different greenspaces. Evidence demonstrates that unstructured outdoor activities and time spent in natural environments can significantly improve children’s mental and physical health, which can plausibly improve sleep [45]. Furthermore, levels of physical activity mediate the relationship between children’s time spent in nature and sleep consistency [46]. Another aspect of nearby park space we could not capture was visual access. Multiple studies have shown that views of nearby nature have restorative properties [12,44,47,48]. Future research should incorporate greenspace exposure, access, and use measurements alongside sleep. Regarding our heat findings, we were not able to measure interior thermal environmental conditions which can influence vulnerability to heat. The presence or absence of bedroom air conditioning, ventilation, and types of bedding materials influence thermal comfort, which in turn influences sleep quality [49]. Interior thermal environments are particularly important during heat wave events, as the highest risk factor for heat-related death is a second-floor bedroom without air conditioning [50]. Future epidemiological research should endeavor to measure interior as well as exterior environmental conditions when examining heat effects on sleep to minimize heat exposure variance. Moreover, we measured extreme heat as the number of heat wave days during the summer months, but we did not have access to other meteorological measures including wind speed, solar radiation, and humidity. Temperature measurements that incorporate ancillary meteorological data such as Heat Index (HI) and Wet Bulb Globe Temperature (WBGT) may provide a more thorough estimation of the heat environment experienced by human beings [51]. Our environmental factors were measured at baseline (year 1), and we assumed children resided in the same address up until year 5. We did not have data on whether children had moved prior to this age. Regarding our sleep measure of multi-year parent-reported sleep measurements for each child, some studies suggest that better measures of sleep exist, such as actigraphy [52]. Finally, a tradeoff to our study’s epidemiological approach is that results are correlational and do not show causal relationships. Future research should consider experimental simulations of heat and natural environment exposure in sleep labs to show causal effects. It is perhaps worth noting, however, that theoretically derived, a priori statistical interaction effects, as those found herein, are less susceptible to most threats to internal validity [53]. Any confounding variable(s) would have to also covary with the interaction of extreme heat and proximity to parks. 

### 4.2. Policy Considerations for Greening in the Context of Climate Change

The frequency, duration, and intensity of heatwaves have increased globally over the last 75 years [54]. Extreme temperatures affect human health and behavior, and negative impacts are projected to increase into the future [55]. Nonetheless we also know that extreme heat has inconsistent impacts within various geographical environments and is differentially experienced based on land-use and built environmental structures [56,57]. Impervious surfaces, such as concrete and asphalt, absorb and retain heat, leading to higher temperatures that are known as the urban heat island effect [58,59]. Conversely, greenspaces with abundant vegetation provide shade and facilitate evaporative cooling, which helps to lower temperatures and mitigate the heat island effect [60,61]. City and regional land use planning must incorporate knowledge of greenspace’s environmental benefits, alongside existing built infrastructure and population characteristics to green children’s neighborhoods. Municipal and city-level partnerships are forming to build nature-filled outdoor spaces [62]. Residents likely possess contextual intelligence on thermal comfort experienced in specific areas that can become critical for making land use changes [63]. The planning process should incorporate children and parents’ nuanced “local knowledge” of thermal needs, leading to the identification of specific sites for greening that otherwise may not be apparent to the institutional planning processes.

## Figures and Tables

**Table 1:** Static variables including Race/Ethnicity, Sex, and Urbanicity show distinct counts of children. Dynamic Variables including combined family income show total observations. Testing Year shows distinct counts per year summing to total observations.

Variable	N/*n*
*Sex*	*8580*
…Male	4488 (52%)
…Female	4092 (48%)
*Race/ethnicity*	*8580*
…White	4642 (54%)
…Black	1139 (13%)
…Hispanic	1690 (20%)
…Asian	206 (2%)
…Other	903 (11%)
*Testing Year*	*33,010*
…Baseline	7945 (24%)
…1 year follow up	7628 (23%)
…2 year follow up	7359 (22%)
…3 year follow up	6835 (21%)
…4 year follow up	3243 (10%)
*Combined Family Income*	*33,010*
…Less than $5000	889 (3%)
…$5000 through $11,999	892 (3%)
…$12,000 through $15,999	617 (2%)
…$16,000 through $24,999	1271 (4%)
…$25,000 through $34,999	1618 (5%)
…$35,000 through $49,999	2325 (7%)
…$50,000 through $74,999	4024 (12%)
…$75,000 through $99,999	4694 (14%)
…$100,000 through $199,999	11,643 (35%)
…$200,000 and greater	5037 (15%)
*Urbanicity*	*8580*
…Urban	7505 (87%)
…Urban Clusters	289 (3%)
…Rural	786 (10%)

**Table 2:** Descriptors of all continuous variables referenced in manuscript.

Variable	Total N	Mean	Median	SD	Minimum	Maximum
Total Sleep Disturbances	33,010	36.30	34.00	7.88	26.00	126.00
Parent Education Level	33,010	17.57	18.00	2.89	1.00	23.00
Age (in Months)	33,010	139.80	139.00	17.81	107.00	189.00
Percent Income to Needs	33,010	432.30	421.08	288.70	5.90	2059.31
Years at Address	33,010	5.85	6.00	3.75	0.00	11.00
Population Density (persons per square mile)	33,010	2116.79	1602.05	2749.71	0.00	60,283.32
Residential Disadvantage Score	33,010	0.10	0.07	0.09	0.01	0.67
Proportion Census Tract Classified as Park Area (in percentage)	33,010	5.00	1.66	8.80	0.00	77.7
Extreme Heat (number of days)	33,010	25.07	15.67	25.97	0.00	135.00

**Table 3:** Main effects and interaction effects on sleep. Results are based on fully adjusted and standardized multilevel model; full model results can be found in Supplemental File S2.

Focal Independent Variables	Effects on Sleep
Proportion Neighborhood Open Park Space	−0.02 *
	(0.01)
Extreme Heat	0.05 **
	(0.02)
Proportion Neighborhood Open Park Space X Extreme Heat	−0.02 *
	(0.01)
Total Observations	33,010
Unique Students	8580
Sites	22
Testing Years	5
Total R^2^	0.65

** *p* < 0.01; * *p* < 0.05.

## Data Availability

The data utilized in this study are from the ABCD cohort and are not publicly available. Access to the data can be requested through the National Institute of Mental Health (NIMH) Data Archive. Researchers interested in accessing the data should apply via the NIMH Data Archive at https://nda.nih.gov/.

## Supplementary Materials

The following supporting information can be downloaded at: https://www.mdpi.com/article/10.3390/ijerph21101270/s1, Supplementary File S1: Shows the flow of data reduction to reach final sample. Data reduction can be conducted in any order to achieve the same final sample; Supplementary File S2: RCode for Neighborhood greenspace, extreme heat exposure, and sleep quality over time along a nationally representative sample of American children.
