# Correction: Rahai et al. Neighborhood Greenspace, Extreme Heat Exposure, and Sleep Quality over Time among a Nationally Representative Sample of American Children. *Int. J. Environ. Res. Public Health* 2024, *21*, 1270

**DOI:** 10.3390/ijerph22040529

**Published:** 2025-03-31

**Authors:** Rouzbeh Rahai, Nancy M. Wells, Gary W. Evans

**Affiliations:** 1 Human Centered Design Department, College of Human Ecology, Cornell University, Ithaca, NY 14850, USA; 2 Psychology Department, College of Human Ecology, Cornell University, Ithaca, NY 14850, USA

In the original publication [1], the following sentence contains an incorrect citation:

“A comprehensive review of heat and sleep finds that ambient indoor and outdoor heat significantly impacts sleep, especially during the hottest days and months worldwide [10]”.

The correct citation should be [9].

The acknowledgments section did not comply with the Data Use Agreement (DUA) for the ABCD Study. Currently, there is not an acknowledgements section. We need to create an acknowledgements section that should read as follows: 

“Data used in the preparation of this article were obtained from the Adolescent Brain Cognitive Development (ABCD) Study (https://abcdstudy.org, accessed on 3 January 2022), held in the NIMH Data Archive (NDA). This is a multisite, longitudinal study designed to recruit more than 10,000 children aged 9–10 years and follow them over 10 years into early adulthood. The ABCD Study^®^ is supported by the National Institutes of Health and additional federal partners under award numbers U01DA041048, U01DA050989, U01DA051016, U01DA041022, U01DA051018, U01DA051037, U01DA050987, U01DA041174, U01DA041106, U01DA041117, U01DA041028, U01DA041134, U01DA050988, U01DA051039, U01DA041156, U01DA041025, U01DA041120, U01DA051038, U01DA041148, U01DA041093, U01DA041089, U24DA041123, and U24DA041147. A full list of supporters is available at https://abcdstudy.org/federal-partners.html, accessed on 3 January 2022. A listing of participating sites and a complete listing of the study investigators can be found at https://abcdstudy.org/consortium_members/, accessed on 3 January 2022. ABCD consortium investigators designed and implemented this study and/or provided data but did not necessarily participate in the analysis or writing of this report. This manuscript reflects the views of the authors and may not reflect the opinions or views of the NIH or ABCD consortium investigators. Additional support for this work was made possible from NIEHS R01-ES032295 and R01-ES031074”.

The authors state that the scientific conclusions are unaffected. This correction was approved by the Academic Editor. The original publication has also been updated.
