# Supplementary material for: Neighborhood Greenspace, Extreme Heat Exposure, and Sleep Quality over Time among a Nationally Representative Sample of American Children
Source: Int J Environ Res Public Health. 2024 Sep 25;21(10):1270. doi: 10.3390/ijerph21101270 (PMC11508033; doi:10.3390/ijerph21101270)
Supplement: Supplementary file 1 [file ijerph-21-01270-s001.zip › ijerph-3152661-supplementary.pdf]

Supplemental File S1

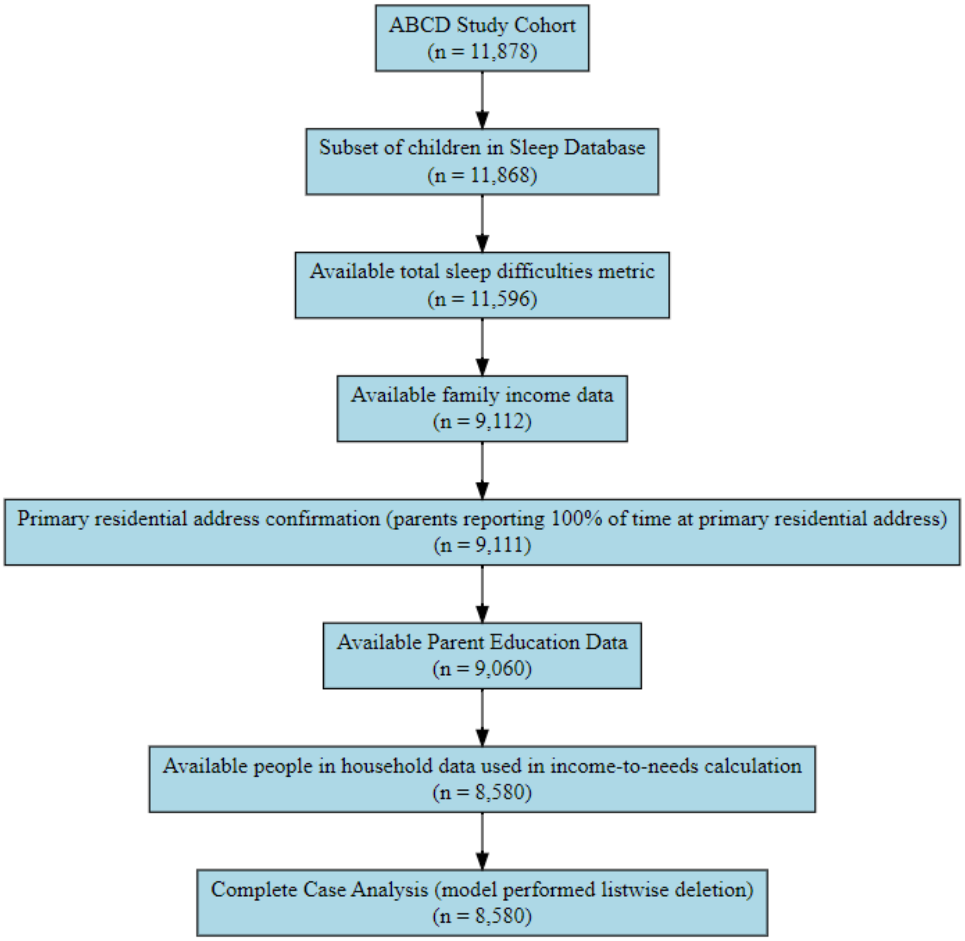

Description: Shows the flow of data reduction to reach final sample. Data reduction can be conducted in any order to achieve the same final sample.

Supplemental File S2: RCode for Neighborhood greenspace, extreme heat exposure, and sleep quality over time among a nationally representative sample of American children

2024-07-26

```
#Extracting Family income, people in household, Parent Education, and Race variables

#Combine family income, parent education, and people in house variables, originally separated, into one populated column
abcd_p_demo_concat <- mutate(abcd_p_demo_concat, Combined_Family_Income = ifelse(is.na(demo_comb_income_v2),
                                                                                 demo_comb_income_v2_l, demo_comb_income_v
2))
abcd_p_demo_concat <- mutate(abcd_p_demo_concat, People_In_House = ifelse(is.na(demo_roster_v2),
                                                                          demo_roster_v2_l, demo_roster_v2))
abcd_p_demo_concat <- abcd_p_demo_concat %>% unite("Parent_Ed_lvl", demo_prnt_ed_v2, demo_prnt_ed_v2_l,
                                                   demo_prnt_ed_v2_2yr_l, sep = "", remove = FALSE, na.rm = TRUE)

#create db of static variables "race_ethnicity" and "sex" at baseline
abcd_p_demo_raceethnicity_sex <- abcd_p_demo_concat %>% filter(eventname=="baseline_year_1_arm_1")
abcd_p_demo_raceethnicity_sex <- abcd_p_demo_raceethnicity_sex %>% dplyr::select(src_subject_id, LongID, eventname, race_eth
nicity, demo_sex_v2)
```

```r
#Creating analytical dataset by selecting relevant variables from merged df

SimpleDataset <- Join28 %>% dplyr::select("src_subject_id.x", "Parent_Ed_lvl", "race_ethnicity.y",
                                          "demo_sex_v2.y", "site_id_l", "interview_age", "eventname.x",
                                          "Combined_Family_Income", "People_In_House", "sds_p_ss_total",
                                          "reshist_addr1_years", "reshist_addr1_popdensity",
                                          "reshist_addr1_nanda_disadv_fac", "reshist_addr1_urban_area",
                                          "reshist_addr1_urbsat_ndvi", "reshist_addr1_coi_he_heat",
                                          "reshist_addr1_percentile", "reshist_addr1_nanda_parks_a_t")



#convert dataset variables to numeric and factors
SimpleDataset_analytical <- within(SimpleDataset, {
  src_subject_id.x <- as.factor(src_subject_id.x)
  Parent_Ed_lvl <- as.numeric(Parent_Ed_lvl)
  race_ethnicity.y <- as.factor(race_ethnicity.y)
  demo_sex_v2.y <- as.numeric(demo_sex_v2.y)
  site_id_l <- as.factor(site_id_l)
  interview_age <- as.numeric(interview_age)
  eventname.x <- as.factor(eventname.x)
  Combined_Family_Income <- as.numeric(Combined_Family_Income)
  People_In_House <- as.numeric(People_In_House)
  sds_p_ss_total <- as.numeric(sds_p_ss_total)
  reshist_addr1_years <- as.numeric(reshist_addr1_years)
  reshist_addr1_popdensity <- as.numeric(reshist_addr1_popdensity)
  reshist_addr1_nanda_disadv_fac <- as.numeric(reshist_addr1_nanda_disadv_fac)
  reshist_addr1_urban_area <- as.numeric(reshist_addr1_urban_area)
  reshist_addr1_urbsat_ndvi <- as.numeric(reshist_addr1_urbsat_ndvi)
  reshist_addr1_coi_he_heat <- as.numeric(reshist_addr1_coi_he_heat)
  reshist_addr1_percentile <- as.numeric(reshist_addr1_percentile)
  reshist_addr1_nanda_parks_a_t <- as.numeric(reshist_addr1_nanda_parks_a_t)
})
```

```
#conducting data reductions, creating income-to-needs, and standardizing variables in model

#filter out null income responses
SimpleDataset_analytical <- SimpleDataset_analytical %>% filter(Combined_Family_Income <777 & Combined_Family_Income > -1)

#filter for only 100% at address 1
SimpleDataset_analytical <- SimpleDataset_analytical %>% filter(reshist_addr1_percentile == 100)

#Filter out null parent Ed
SimpleDataset_analytical <- SimpleDataset_analytical %>% filter(Parent_Ed_lvl < 777 & Parent_Ed_lvl > -1)

#Filter out atypical people in houses response
SimpleDataset_analytical <- SimpleDataset_analytical %>% filter(People_In_House < 40 & People_In_House > 0)

#Filter out atypical people in houses response
SimpleDataset_analytical <- SimpleDataset_analytical %>% filter(demo_sex_v2.y < 3)


#Recode Family income as median
SimpleDataset_analytical$Combined_Family_Income_recode <- recode(SimpleDataset_analytical$Combined_Family_Income, "1=2500; 2
=8500; 3=14000; 4=20500; 5=30000; 6=42500;
                                                          7=62500; 8=87500; 9=150000; 10=250000")


#Assign FPL threshold based on number of people in house
SimpleDataset_analytical <- SimpleDataset_analytical %>% dplyr::mutate(FPL = case_when(People_In_House == 1 ~ 12140,
                                                                                       People_In_House == 2 ~ 16460,
                                                                                       People_In_House == 3 ~ 20780,
                                                                                       People_In_House == 4 ~ 25100,
                                                                                       People_In_House == 5 ~ 29420,
                                                                                       People_In_House == 6 ~ 33740,
                                                                                       People_In_House == 7 ~ 38060,
                                                                                       People_In_House > 7 ~ 42380))

#new varibales as numeric
SimpleDataset_analytical <- within( SimpleDataset_analytical, {
  Combined_Family_Income_recode <- as.numeric(Combined_Family_Income_recode)
  FPL <- as.numeric(FPL)
} )

#calculate estimated income to needs
```

```
SimpleDataset_analytical <- SimpleDataset_analytical %>% dplyr::mutate (income_to_needs = (Combined_Family_Income_recode/FP
L)*100 )


#income_to_needs as numeric
SimpleDataset_analytical <- within( SimpleDataset_analytical, {
  income_to_needs <- as.numeric(income_to_needs)
} )

SimpleDataset_analytical <- within(SimpleDataset_analytical, {
  src_subject_id.x <- as.factor(src_subject_id.x)
  Parent_Ed_lvl <- as.numeric(Parent_Ed_lvl)
  race_ethnicity.y <- as.factor(race_ethnicity.y)
  demo_sex_v2.y <- as.factor(demo_sex_v2.y)
  site_id_l <- as.factor(site_id_l)
  interview_age <- as.numeric(interview_age)
  eventname.x <- as.factor(eventname.x)
  Combined_Family_Income <- as.numeric(Combined_Family_Income)
  People_In_House <- as.numeric(People_In_House)
  sds_p_ss_total <- as.numeric(sds_p_ss_total)
  reshist_addr1_years <- as.numeric(reshist_addr1_years)
  reshist_addr1_popdensity <- as.numeric(reshist_addr1_popdensity)
  reshist_addr1_nanda_disadv_fac <- as.numeric(reshist_addr1_nanda_disadv_fac)
  reshist_addr1_urban_area <- as.factor(reshist_addr1_urban_area)
  reshist_addr1_urbsat_ndvi <- as.numeric(reshist_addr1_urbsat_ndvi)
  reshist_addr1_coi_he_heat <- as.numeric(reshist_addr1_coi_he_heat)
  reshist_addr1_percentile <- as.numeric(reshist_addr1_percentile)
  reshist_addr1_nanda_parks_a_t <- as.numeric(reshist_addr1_nanda_parks_a_t)
})

SimpleDataset_analytical_z <- within(SimpleDataset_analytical, {
  Parent_Ed_lvl <- scale(Parent_Ed_lvl)
  interview_age <- scale(interview_age)
  income_to_needs <- scale(income_to_needs)
  sds_p_ss_total <- scale(sds_p_ss_total)
  reshist_addr1_popdensity <- scale(reshist_addr1_popdensity)
  reshist_addr1_nanda_disadv_fac <- scale(reshist_addr1_nanda_disadv_fac)
  reshist_addr1_urbsat_ndvi <- scale(reshist_addr1_urbsat_ndvi)
  reshist_addr1_coi_he_heat <- scale(reshist_addr1_coi_he_heat)
  reshist_addr1_nanda_parks_a_t <- scale(reshist_addr1_nanda_parks_a_t)
})
```

```r
#Running multilevel model and creating table output

Test1 <- lmer(sds_p_ss_total ~  Parent_Ed_lvl + race_ethnicity.y  + demo_sex_v2.y + interview_age +
                income_to_needs + reshist_addr1_years + reshist_addr1_popdensity + reshist_addr1_nanda_disadv_fac +
                reshist_addr1_urban_area +
                reshist_addr1_nanda_parks_a_t*reshist_addr1_coi_he_heat +
                (1|src_subject_id.x) + (1|eventname.x) + (1|site_id_l), data=SimpleDataset_analytical_z)

vif(Test1)
```

```
##                                                             GVIF Df
## Parent_Ed_lvl                                           1.062049  1
## race_ethnicity.y                                        1.601650  4
## demo_sex_v2.y                                           1.001056  1
## interview_age                                           1.005822  1
## income_to_needs                                         1.191605  1
## reshist_addr1_years                                     1.055668  1
## reshist_addr1_popdensity                                1.099797  1
## reshist_addr1_nanda_disadv_fac                          1.624910  1
## reshist_addr1_urban_area                                1.070494  2
## reshist_addr1_nanda_parks_a_t                           1.072775  1
## reshist_addr1_coi_he_heat                               1.019811  1
## reshist_addr1_nanda_parks_a_t:reshist_addr1_coi_he_heat 1.072464  1
##                                                         GVIF^(1/(2*Df))
## Parent_Ed_lvl                                                  1.030557
## race_ethnicity.y                                               1.060647
## demo_sex_v2.y                                                  1.000528
## interview_age                                                  1.002907
## income_to_needs                                                1.091607
## reshist_addr1_years                                            1.027457
## reshist_addr1_popdensity                                       1.048712
## reshist_addr1_nanda_disadv_fac                                 1.274720
## reshist_addr1_urban_area                                       1.017176
## reshist_addr1_nanda_parks_a_t                                  1.035748
## reshist_addr1_coi_he_heat                                      1.009857
## reshist_addr1_nanda_parks_a_t:reshist_addr1_coi_he_heat        1.035598
```

```r
summary(Test1)
```

```
## Linear mixed model fit by REML. t-tests use Satterthwaite's method [
## lmerModLmerTest]
## Formula: sds_p_ss_total ~ Parent_Ed_lvl + race_ethnicity.y + demo_sex_v2.y +
##     interview_age + income_to_needs + reshist_addr1_years + reshist_addr1_popdensity +
##     reshist_addr1_nanda_disadv_fac + reshist_addr1_urban_area +
##     reshist_addr1_nanda_parks_a_t * reshist_addr1_coi_he_heat +
##     (1 | src_subject_id.x) + (1 | eventname.x) + (1 | site_id_l)
##    Data: SimpleDataset_analytical_z
##
## REML criterion at convergence: 76980
##
## Scaled residuals:
##     Min      1Q  Median      3Q     Max
## -6.3212 -0.4979 -0.1062  0.4031 14.2600
##
## Random effects:
##  Groups           Name        Variance Std.Dev.
##  src_subject_id.x (Intercept) 0.63432  0.79644
##  site_id_l        (Intercept) 0.01227  0.11075
##  eventname.x      (Intercept) 0.00103  0.03209
##  Residual                     0.35537  0.59613
## Number of obs: 33010, groups:
## src_subject_id.x, 8580; site_id_l, 22; eventname.x, 5
##
## Fixed effects:
##                                                         Estimate Std. Error
## (Intercept)                                            3.918e-02  3.685e-02
## Parent_Ed_lvl                                         -6.109e-03  5.778e-03
## race_ethnicity.y2                                     -4.358e-02  3.735e-02
## race_ethnicity.y3                                     -4.132e-02  2.979e-02
## race_ethnicity.y4                                     -7.268e-02  6.341e-02
## race_ethnicity.y5                                      1.075e-01  3.238e-02
## demo_sex_v2.y2                                        -2.058e-02  1.861e-02
## interview_age                                         -1.905e-02  1.185e-02
## income_to_needs                                       -1.885e-02  8.200e-03
## reshist_addr1_years                                   -6.439e-03  2.578e-03
## reshist_addr1_popdensity                               1.301e-02  1.011e-02
## reshist_addr1_nanda_disadv_fac                         3.721e-02  1.236e-02
## reshist_addr1_urban_area2                              5.361e-02  5.311e-02
## reshist_addr1_urban_area3                              3.646e-02  3.509e-02
## reshist_addr1_nanda_parks_a_t                         -1.915e-02  9.697e-03
```

```
## reshist_addr1_coi_he_heat                                4.891e-02  1.727e-02
## reshist_addr1_nanda_parks_a_t:reshist_addr1_coi_he_heat -2.210e-02  1.073e-02
##                                                                 df t value
## (Intercept)                                              4.148e+01   1.063
## Parent_Ed_lvl                                            3.090e+04  -1.057
## race_ethnicity.y2                                        8.776e+03  -1.167
## race_ethnicity.y3                                        5.987e+03  -1.387
## race_ethnicity.y4                                        8.182e+03  -1.146
## race_ethnicity.y5                                        8.384e+03   3.321
## demo_sex_v2.y2                                           8.405e+03  -1.106
## interview_age                                            6.224e+00  -1.608
## income_to_needs                                          2.765e+04  -2.299
## reshist_addr1_years                                      8.484e+03  -2.498
## reshist_addr1_popdensity                                 6.974e+03   1.287
## reshist_addr1_nanda_disadv_fac                           8.287e+03   3.009
## reshist_addr1_urban_area2                                8.314e+03   1.009
## reshist_addr1_urban_area3                                7.934e+03   1.039
## reshist_addr1_nanda_parks_a_t                            8.567e+03  -1.975
## reshist_addr1_coi_he_heat                                2.095e+02   2.832
## reshist_addr1_nanda_parks_a_t:reshist_addr1_coi_he_heat  8.744e+03  -2.060
##                                                         Pr(>|t|)
## (Intercept)                                              0.29380
## Parent_Ed_lvl                                            0.29040
## race_ethnicity.y2                                        0.24334
## race_ethnicity.y3                                        0.16556
## race_ethnicity.y4                                        0.25175
## race_ethnicity.y5                                        0.00090 ***
## demo_sex_v2.y2                                           0.26891
## interview_age                                            0.15715
## income_to_needs                                          0.02152 *
## reshist_addr1_years                                      0.01252 *
## reshist_addr1_popdensity                                 0.19818
## reshist_addr1_nanda_disadv_fac                           0.00263 **
## reshist_addr1_urban_area2                                0.31282
## reshist_addr1_urban_area3                                0.29881
## reshist_addr1_nanda_parks_a_t                            0.04831 *
## reshist_addr1_coi_he_heat                                0.00508 **
## reshist_addr1_nanda_parks_a_t:reshist_addr1_coi_he_heat  0.03941 *
## ---
## Signif. codes:  0 '***' 0.001 '**' 0.01 '*' 0.05 '.' 0.1 ' ' 1
```

```
##
## Correlation matrix not shown by default, as p = 17 > 12.
## Use print(x, correlation=TRUE)  or
##     vcov(x)        if you need it
```

```
model_performance(Test1)
```

```
## # Indices of model performance
##
## AIC       |      AICc |       BIC | R2 (cond.) | R2 (marg.) |   ICC |  RMSE | Sigma
## ---------------------------------------------------------------------------------
## 77022.028 | 77022.056 | 77198.524 |      0.649 |      0.010 | 0.646 | 0.525 | 0.596
```

```
jtools::export_summs(Test1, scale = FALSE, to.file = "xlsx", file.name = "FinalTable_1.xlsx")
```

```
## If summ is taking too long to run, try setting r.squared = FALSE.
```

	Model 1
(Intercept)	0.04
	(0.04)
Parent_Ed_lvl	-0.01
	(0.01)
race_ethnicity.y2	-0.04
	(0.04)
race_ethnicity.y3	-0.04
	(0.03)
race_ethnicity.y4	-0.07

	(0.06)
race_ethnicity.y5	0.11 ***
	(0.03)
demo_sex_v2.y2	-0.02
	(0.02)
interview_age	-0.02
	(0.01)
income_to_needs	-0.02 *
	(0.01)
reshist_addr1_years	-0.01 *
	(0.00)
reshist_addr1_popdensity	0.01
	(0.01)
reshist_addr1_nanda_disadv_fac	0.04 **
	(0.01)
reshist_addr1_urban_area2	0.05
	(0.05)
reshist_addr1_urban_area3	0.04
	(0.04)
reshist_addr1_nanda_parks_a_t	-0.02 *
	(0.01)

reshist_addr1_coi_he_heat	0.05 **
	(0.02)
reshist_addr1_nanda_parks_a_t:reshist_addr1_coi_he_heat	-0.02 *
	(0.01)
N	33010
N (src_subject_id.x)	8580
N (site_id_l)	22
N (eventname.x)	5
AIC	77022.03
BIC	77198.52
R2 (fixed)	0.01
R2 (total)	0.65

*** p < 0.001; ** p < 0.01; * p < 0.05.

```
#plotting interaction results

# Create the effect plot
ParksXHeat <- effect(term="reshist_addr1_nanda_parks_a_t*reshist_addr1_coi_he_heat",
                     xlevels= list(reshist_addr1_nanda_parks_a_t=c(-1,0,1),
                                   reshist_addr1_coi_he_heat=c(-3,0,3)), mod = Test1)
```

```
## Warning in Analyze.model(focal.predictors, mod, xlevels, default.levels, : the
## predictors sds_p_ss_total, Parent_Ed_lvl, interview_age, income_to_needs,
## reshist_addr1_popdensity, reshist_addr1_nanda_disadv_fac,
## reshist_addr1_nanda_parks_a_t, reshist_addr1_coi_he_heat are one-column
## matrices that were converted to vectors
```

```r
ParksXHeat <- as.data.frame(ParksXHeat)

ParksXHeat$reshist_addr1_nanda_parks_a_t <- as.factor(ParksXHeat$reshist_addr1_nanda_parks_a_t)

plot1A <- ggplot(ParksXHeat, aes(x=reshist_addr1_coi_he_heat, y=fit, color=reshist_addr1_nanda_parks_a_t, group=reshist_addr
1_nanda_parks_a_t)) +
  geom_point() +
  geom_line(size=1.2) +
  geom_ribbon(aes(ymin=fit, ymax=fit, fill=reshist_addr1_nanda_parks_a_t), alpha=0.3) +
  labs(title = "", x= "Neighborhood Extreme Heat", y="Sleep Disturbances",
       color="Park Area", fill="Park Area") + theme_classic() + theme(text=element_text(size=20))
```

```
## Warning: Using `size` aesthetic for lines was deprecated in ggplot2 3.4.0.
## ℹ Please use `linewidth` instead.
## This warning is displayed once every 8 hours.
## Call `lifecycle::last_lifecycle_warnings()` to see where this warning was
## generated.
```

```r
# Print the plot
print(plot1A)
```

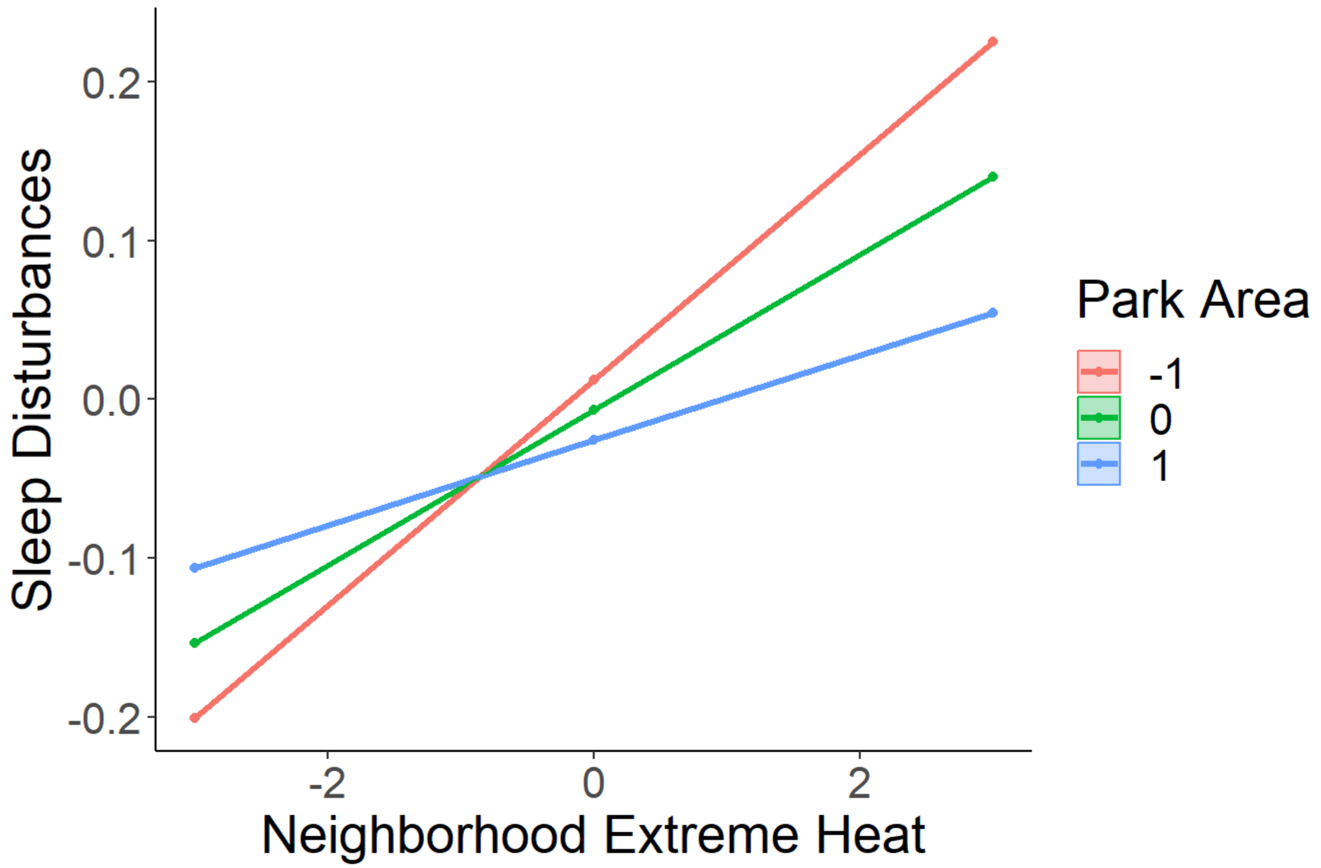


```
  #Sensitivity analysis and model building

##Additive interaction model
Test2 <- lmer(sds_p_ss_total ~ Parent_Ed_lvl + race_ethnicity.y + demo_sex_v2.y + interview_age +
                income_to_needs + reshist_addr1_years + reshist_addr1_popdensity + reshist_addr1_nanda_disadv_fac +
                reshist_addr1_urban_area +
                I(reshist_addr1_nanda_parks_a_t + reshist_addr1_coi_he_heat) +
                (1|src_subject_id.x) + (1|eventname.x) + (1|site_id_l), data=SimpleDataset_analytical_z)
vif(Test2)
```

```
##                                                                   GVIF Df
## Parent_Ed_lvl                                                 1.061714  1
## race_ethnicity.y                                              1.593888  4
## demo_sex_v2.y                                                 1.000967  1
## interview_age                                                 1.005799  1
## income_to_needs                                               1.187129  1
## reshist_addr1_years                                           1.054846  1
## reshist_addr1_popdensity                                      1.096336  1
## reshist_addr1_nanda_disadv_fac                                1.619337  1
## reshist_addr1_urban_area                                      1.061686  2
## I(reshist_addr1_nanda_parks_a_t + reshist_addr1_coi_he_heat) 1.011511  1
##                                                               GVIF^(1/(2*Df))
## Parent_Ed_lvl                                                        1.030395
## race_ethnicity.y                                                     1.060003
## demo_sex_v2.y                                                        1.000483
## interview_age                                                        1.002895
## income_to_needs                                                      1.089555
## reshist_addr1_years                                                  1.027057
## reshist_addr1_popdensity                                             1.047061
## reshist_addr1_nanda_disadv_fac                                       1.272532
## reshist_addr1_urban_area                                             1.015077
## I(reshist_addr1_nanda_parks_a_t + reshist_addr1_coi_he_heat)        1.005739
```

```
summary(Test2)
```

```
## Linear mixed model fit by REML. t-tests use Satterthwaite's method [
## lmerModLmerTest]
## Formula: sds_p_ss_total ~ Parent_Ed_lvl + race_ethnicity.y + demo_sex_v2.y +
##     interview_age + income_to_needs + reshist_addr1_years + reshist_addr1_popdensity +
##     reshist_addr1_nanda_disadv_fac + reshist_addr1_urban_area +
##     I(reshist_addr1_nanda_parks_a_t + reshist_addr1_coi_he_heat) +
##     (1 | src_subject_id.x) + (1 | eventname.x) + (1 | site_id_l)
##    Data: SimpleDataset_analytical_z
##
## REML criterion at convergence: 76982.4
##
## Scaled residuals:
##     Min      1Q  Median      3Q     Max
## -6.3067 -0.4977 -0.1063  0.4031 14.2626
##
## Random effects:
##  Groups           Name        Variance Std.Dev.
##  src_subject_id.x (Intercept) 0.635355 0.79709
##  site_id_l        (Intercept) 0.014058 0.11857
##  eventname.x      (Intercept) 0.001008 0.03175
##  Residual                     0.355365 0.59613
## Number of obs: 33010, groups:
## src_subject_id.x, 8580; site_id_l, 22; eventname.x, 5
##
## Fixed effects:
##                                                                 Estimate
## (Intercept)                                                    3.918e-02
## Parent_Ed_lvl                                                 -6.171e-03
## race_ethnicity.y2                                             -4.326e-02
## race_ethnicity.y3                                             -4.055e-02
## race_ethnicity.y4                                             -7.117e-02
## race_ethnicity.y5                                              1.085e-01
## demo_sex_v2.y2                                                -1.990e-02
## interview_age                                                 -1.941e-02
## income_to_needs                                               -2.035e-02
## reshist_addr1_years                                           -6.637e-03
## reshist_addr1_popdensity                                       1.143e-02
## reshist_addr1_nanda_disadv_fac                                 3.709e-02
## reshist_addr1_urban_area2                                      5.938e-02
## reshist_addr1_urban_area3                                      4.760e-02
## I(reshist_addr1_nanda_parks_a_t + reshist_addr1_coi_he_heat) -7.682e-04
```

```
##                                                         Std. Error
## (Intercept)                                              3.789e-02
## Parent_Ed_lvl                                            5.778e-03
## race_ethnicity.y2                                        3.737e-02
## race_ethnicity.y3                                        2.985e-02
## race_ethnicity.y4                                        6.347e-02
## race_ethnicity.y5                                        3.240e-02
## demo_sex_v2.y2                                           1.863e-02
## interview_age                                            1.178e-02
## income_to_needs                                          8.193e-03
## reshist_addr1_years                                      2.579e-03
## reshist_addr1_popdensity                                 1.012e-02
## reshist_addr1_nanda_disadv_fac                           1.237e-02
## reshist_addr1_urban_area2                                5.312e-02
## reshist_addr1_urban_area3                                3.503e-02
## I(reshist_addr1_nanda_parks_a_t + reshist_addr1_coi_he_heat)  8.517e-03
##                                                                 df t value
## (Intercept)                                              4.229e+01   1.034
## Parent_Ed_lvl                                            3.088e+04  -1.068
## race_ethnicity.y2                                        8.782e+03  -1.158
## race_ethnicity.y3                                        6.438e+03  -1.358
## race_ethnicity.y4                                        8.260e+03  -1.121
## race_ethnicity.y5                                        8.389e+03   3.348
## demo_sex_v2.y2                                           8.404e+03  -1.068
## interview_age                                            6.239e+00  -1.648
## income_to_needs                                          2.754e+04  -2.484
## reshist_addr1_years                                      8.487e+03  -2.573
## reshist_addr1_popdensity                                 6.970e+03   1.129
## reshist_addr1_nanda_disadv_fac                           8.466e+03   2.998
## reshist_addr1_urban_area2                                8.342e+03   1.118
## reshist_addr1_urban_area3                                8.000e+03   1.359
## I(reshist_addr1_nanda_parks_a_t + reshist_addr1_coi_he_heat)  3.414e+03  -0.090
##                                                         Pr(>|t|)
## (Intercept)                                             0.306999
## Parent_Ed_lvl                                           0.285575
## race_ethnicity.y2                                       0.247045
## race_ethnicity.y3                                       0.174407
## race_ethnicity.y4                                       0.262132
## race_ethnicity.y5                                       0.000816 ***
## demo_sex_v2.y2                                          0.285463
## interview_age                                           0.148547
```

```
## income_to_needs                                        0.013009 *
## reshist_addr1_years                                    0.010095 *
## reshist_addr1_popdensity                               0.259026
## reshist_addr1_nanda_disadv_fac                         0.002730 **
## reshist_addr1_urban_area2                              0.263683
## reshist_addr1_urban_area3                              0.174222
## I(reshist_addr1_nanda_parks_a_t + reshist_addr1_coi_he_heat) 0.928136
## ---
## Signif. codes:  0 '***' 0.001 '**' 0.01 '*' 0.05 '.' 0.1 ' ' 1
```

```
##
## Correlation matrix not shown by default, as p = 15 > 12.
## Use print(x, correlation=TRUE)  or
##     vcov(x)        if you need it
```

```
model_performance(Test2)
```

```
## # Indices of model performance
##
## AIC       |      AICc |       BIC | R2 (cond.) | R2 (marg.) |   ICC |  RMSE | Sigma
## ---------------------------------------------------------------------------------
## 77020.437 | 77020.460 | 77180.124 |      0.649 |      0.006 | 0.647 | 0.525 | 0.596
```

```
##Sensitivity analysis based on skewness tests and IQR-transformed variables
skewness(SimpleDataset_analytical$reshist_addr1_coi_he_heat, na.rm = TRUE)
```

```
## [1] 1.337141
```

```
skewness(SimpleDataset_analytical$reshist_addr1_nanda_parks_a_t, na.rm = TRUE)
```

```
## [1] 3.339419
```

```
skewness(SimpleDataset_analytical$sds_p_ss_total, na.rm = TRUE)
```

```
## [1] 1.666072
```

```
skewness(SimpleDataset_analytical$interview_age, na.rm = TRUE)
```

```
## [1] 0.1810557
```

```
skewness(SimpleDataset_analytical$income_to_needs, na.rm = TRUE)
```

```
## [1] 0.5999301
```

```
skewness(SimpleDataset_analytical$reshist_addr1_years, na.rm = TRUE)
```

```
## [1] -0.09464575
```

```
skewness(SimpleDataset_analytical$reshist_addr1_popdensity, na.rm = TRUE)
```

```
## [1] 8.544953
```

```
skewness(SimpleDataset_analytical$reshist_addr1_nanda_disadv_fac, na.rm = TRUE)
```

```
## [1] 2.191243
```

```
# IQR-based transformation for skewed variables
SimpleDataset_analytical$reshist_addr1_coi_he_heat_IQR <- cut(SimpleDataset_analytical$reshist_addr1_coi_he_heat,
                                                              quantile(SimpleDataset_analytical$reshist_addr1_coi_he_heat,
                                                                       probs = seq(0, 1, by = 0.25), na.rm = TRUE),
                                                              include.lowest = TRUE)

SimpleDataset_analytical$reshist_addr1_nanda_parks_a_t_IQR <- cut(SimpleDataset_analytical$reshist_addr1_nanda_parks_a_t,
                                                                  quantile(SimpleDataset_analytical$reshist_addr1_nanda_park
s_a_t,
                                                                           probs = seq(0, 1, by = 0.25), na.rm = TRUE),
                                                                  include.lowest = TRUE)

SimpleDataset_analytical$reshist_addr1_popdensity_IQR <- cut(SimpleDataset_analytical$reshist_addr1_popdensity,
                                                             quantile(SimpleDataset_analytical$reshist_addr1_popdensity,
                                                                      probs = seq(0, 1, by = 0.25), na.rm = TRUE),
                                                             include.lowest = TRUE)

SimpleDataset_analytical$reshist_addr1_nanda_disadv_fac_IQR <- cut(SimpleDataset_analytical$reshist_addr1_nanda_disadv_fac,
                                                                   quantile(SimpleDataset_analytical$reshist_addr1_nanda_dis
adv_fac,
                                                                            probs = seq(0, 1, by = 0.25), na.rm = TRUE),
                                                                   include.lowest = TRUE)

# Run the model with IQR-transformed variables
Test_IQR <- lmer(sds_p_ss_total ~ Parent_Ed_lvl + race_ethnicity.y + demo_sex_v2.y + interview_age +
                   income_to_needs  + reshist_addr1_years + reshist_addr1_popdensity_IQR +
                   reshist_addr1_nanda_disadv_fac_IQR + reshist_addr1_urban_area +
                   reshist_addr1_nanda_parks_a_t_IQR * reshist_addr1_coi_he_heat_IQR +
                   (1|src_subject_id.x) + (1|eventname.x) + (1|site_id_l),
                 data=SimpleDataset_analytical)

summary(Test_IQR)
```

```
## Linear mixed model fit by REML. t-tests use Satterthwaite's method [
## lmerModLmerTest]
## Formula: sds_p_ss_total ~ Parent_Ed_lvl + race_ethnicity.y + demo_sex_v2.y +
##     interview_age + income_to_needs + reshist_addr1_years + reshist_addr1_popdensity_IQR +
##     reshist_addr1_nanda_disadv_fac_IQR + reshist_addr1_urban_area +
##     reshist_addr1_nanda_parks_a_t_IQR * reshist_addr1_coi_he_heat_IQR +
##     (1 | src_subject_id.x) + (1 | eventname.x) + (1 | site_id_l)
##    Data: SimpleDataset_analytical
##
## REML criterion at convergence: 213257
##
## Scaled residuals:
##     Min      1Q  Median      3Q     Max
## -6.3337 -0.4983 -0.1057  0.4033 14.2526
##
## Random effects:
##  Groups           Name        Variance Std.Dev.
##  src_subject_id.x (Intercept) 39.50436 6.2852
##  site_id_l        (Intercept)  0.85055 0.9223
##  eventname.x      (Intercept)  0.06343 0.2519
##  Residual                     22.09811 4.7009
## Number of obs: 33010, groups:
## src_subject_id.x, 8580; site_id_l, 22; eventname.x, 5
##
## Fixed effects:
##                                                                          Estimate
## (Intercept)                                                             3.764e+01
## Parent_Ed_lvl                                                          -1.942e-02
## race_ethnicity.y2                                                      -1.386e-01
## race_ethnicity.y3                                                      -2.911e-01
## race_ethnicity.y4                                                      -6.115e-01
## race_ethnicity.y5                                                       8.855e-01
## demo_sex_v2.y2                                                         -1.737e-01
## interview_age                                                          -8.553e-03
## income_to_needs                                                        -5.083e-04
## reshist_addr1_years                                                    -5.528e-02
## reshist_addr1_popdensity_IQR(776,1.61e+03]                              3.283e-01
## reshist_addr1_popdensity_IQR(1.61e+03,2.69e+03]                         5.119e-01
## reshist_addr1_popdensity_IQR(2.69e+03,6.03e+04]                         1.291e-01
## reshist_addr1_nanda_disadv_fac_IQR(0.0394,0.0687]                       1.964e-01
## reshist_addr1_nanda_disadv_fac_IQR(0.0687,0.125]                        4.915e-01
```

```
 # reshist_addr1_nanda_disadv_fac_IQR(0.125,0.67]                                                    7.257e-01
## reshist_addr1_urban_area2                                                                         5.208e-01
## reshist_addr1_urban_area3                                                                         4.409e-01
## reshist_addr1_nanda_parks_a_t_IQR(0.000814,0.0166]                                               -1.504e-01
## reshist_addr1_nanda_parks_a_t_IQR(0.0166,0.0556]                                                 -1.259e-02
## reshist_addr1_nanda_parks_a_t_IQR(0.0556,0.766]                                                  -3.769e-02
## reshist_addr1_coi_he_heat_IQR(6,15.7]                                                             6.433e-03
## reshist_addr1_coi_he_heat_IQR(15.7,36]                                                            4.504e-01
## reshist_addr1_coi_he_heat_IQR(36,135]                                                             9.335e-01
## reshist_addr1_nanda_parks_a_t_IQR(0.000814,0.0166]:reshist_addr1_coi_he_heat_IQR(6,15.7]  -5.732e-02
## reshist_addr1_nanda_parks_a_t_IQR(0.0166,0.0556]:reshist_addr1_coi_he_heat_IQR(6,15.7]     1.534e-01
## reshist_addr1_nanda_parks_a_t_IQR(0.0556,0.766]:reshist_addr1_coi_he_heat_IQR(6,15.7]      2.630e-01
## reshist_addr1_nanda_parks_a_t_IQR(0.000814,0.0166]:reshist_addr1_coi_he_heat_IQR(15.7,36]  3.182e-01
## reshist_addr1_nanda_parks_a_t_IQR(0.0166,0.0556]:reshist_addr1_coi_he_heat_IQR(15.7,36]   -4.399e-01
## reshist_addr1_nanda_parks_a_t_IQR(0.0556,0.766]:reshist_addr1_coi_he_heat_IQR(15.7,36]    -7.458e-01
## reshist_addr1_nanda_parks_a_t_IQR(0.000814,0.0166]:reshist_addr1_coi_he_heat_IQR(36,135]  -4.694e-01
## reshist_addr1_nanda_parks_a_t_IQR(0.0166,0.0556]:reshist_addr1_coi_he_heat_IQR(36,135]    -8.905e-01
## reshist_addr1_nanda_parks_a_t_IQR(0.0556,0.766]:reshist_addr1_coi_he_heat_IQR(36,135]     -9.347e-01
##                                                                                          Std. Error
## (Intercept)                                                                               9.234e-01
## Parent_Ed_lvl                                                                             1.557e-02
## race_ethnicity.y2                                                                         2.768e-01
## race_ethnicity.y3                                                                         2.381e-01
## race_ethnicity.y4                                                                         5.017e-01
## race_ethnicity.y5                                                                         2.553e-01
## demo_sex_v2.y2                                                                            1.471e-01
## interview_age                                                                             5.236e-03
## income_to_needs                                                                           2.259e-04
## reshist_addr1_years                                                                       2.038e-02
## reshist_addr1_popdensity_IQR(776,1.61e+03]                                                2.439e-01
## reshist_addr1_popdensity_IQR(1.61e+03,2.69e+03]                                           2.600e-01
## reshist_addr1_popdensity_IQR(2.69e+03,6.03e+04]                                           2.857e-01
## reshist_addr1_nanda_disadv_fac_IQR(0.0394,0.0687]                                         2.173e-01
## reshist_addr1_nanda_disadv_fac_IQR(0.0687,0.125]                                          2.332e-01
## reshist_addr1_nanda_disadv_fac_IQR(0.125,0.67]                                            2.681e-01
## reshist_addr1_urban_area2                                                                 4.335e-01
## reshist_addr1_urban_area3                                                                 3.208e-01
## reshist_addr1_nanda_parks_a_t_IQR(0.000814,0.0166]                                        4.272e-01
## reshist_addr1_nanda_parks_a_t_IQR(0.0166,0.0556]                                          4.265e-01
## reshist_addr1_nanda_parks_a_t_IQR(0.0556,0.766]                                           4.220e-01
## reshist_addr1_coi_he_heat_IQR(6,15.7]                                                     4.748e-01
```

```
 # reshist_addr1_coi_he_heat_IQR(15.7,36]                                           4.457e-01
## reshist_addr1_coi_he_heat_IQR(36,135]                                            4.837e-01
## reshist_addr1_nanda_parks_a_t_IQR(0.000814,0.0166]:reshist_addr1_coi_he_heat_IQR(6,15.7]   6.105e-01
## reshist_addr1_nanda_parks_a_t_IQR(0.0166,0.0556]:reshist_addr1_coi_he_heat_IQR(6,15.7]     6.108e-01
## reshist_addr1_nanda_parks_a_t_IQR(0.0556,0.766]:reshist_addr1_coi_he_heat_IQR(6,15.7]      5.962e-01
## reshist_addr1_nanda_parks_a_t_IQR(0.000814,0.0166]:reshist_addr1_coi_he_heat_IQR(15.7,36]  5.979e-01
## reshist_addr1_nanda_parks_a_t_IQR(0.0166,0.0556]:reshist_addr1_coi_he_heat_IQR(15.7,36]    5.962e-01
## reshist_addr1_nanda_parks_a_t_IQR(0.0556,0.766]:reshist_addr1_coi_he_heat_IQR(15.7,36]     5.790e-01
## reshist_addr1_nanda_parks_a_t_IQR(0.000814,0.0166]:reshist_addr1_coi_he_heat_IQR(36,135]   5.825e-01
## reshist_addr1_nanda_parks_a_t_IQR(0.0166,0.0556]:reshist_addr1_coi_he_heat_IQR(36,135]     5.879e-01
## reshist_addr1_nanda_parks_a_t_IQR(0.0556,0.766]:reshist_addr1_coi_he_heat_IQR(36,135]      6.324e-01
##                                                                                                    df
## (Intercept)                                                                         1.252e+01
## Parent_Ed_lvl                                                                       3.089e+04
## race_ethnicity.y2                                                                   8.781e+03
## race_ethnicity.y3                                                                   6.834e+03
## race_ethnicity.y4                                                                   8.241e+03
## race_ethnicity.y5                                                                   8.378e+03
## demo_sex_v2.y2                                                                      8.389e+03
## interview_age                                                                       6.275e+00
## income_to_needs                                                                     2.797e+04
## reshist_addr1_years                                                                 8.484e+03
## reshist_addr1_popdensity_IQR(776,1.61e+03]                                          8.296e+03
## reshist_addr1_popdensity_IQR(1.61e+03,2.69e+03]                                     7.894e+03
## reshist_addr1_popdensity_IQR(2.69e+03,6.03e+04]                                     6.431e+03
## reshist_addr1_nanda_disadv_fac_IQR(0.0394,0.0687]                                   8.243e+03
## reshist_addr1_nanda_disadv_fac_IQR(0.0687,0.125]                                    8.055e+03
## reshist_addr1_nanda_disadv_fac_IQR(0.125,0.67]                                      8.025e+03
## reshist_addr1_urban_area2                                                           8.337e+03
## reshist_addr1_urban_area3                                                           8.290e+03
## reshist_addr1_nanda_parks_a_t_IQR(0.000814,0.0166]                                  8.496e+03
## reshist_addr1_nanda_parks_a_t_IQR(0.0166,0.0556]                                    8.472e+03
## reshist_addr1_nanda_parks_a_t_IQR(0.0556,0.766]                                     8.416e+03
## reshist_addr1_coi_he_heat_IQR(6,15.7]                                               6.266e+03
## reshist_addr1_coi_he_heat_IQR(15.7,36]                                              6.548e+03
## reshist_addr1_coi_he_heat_IQR(36,135]                                               1.498e+03
## reshist_addr1_nanda_parks_a_t_IQR(0.000814,0.0166]:reshist_addr1_coi_he_heat_IQR(6,15.7]   8.460e+03
## reshist_addr1_nanda_parks_a_t_IQR(0.0166,0.0556]:reshist_addr1_coi_he_heat_IQR(6,15.7]     8.457e+03
## reshist_addr1_nanda_parks_a_t_IQR(0.0556,0.766]:reshist_addr1_coi_he_heat_IQR(6,15.7]      8.430e+03
## reshist_addr1_nanda_parks_a_t_IQR(0.000814,0.0166]:reshist_addr1_coi_he_heat_IQR(15.7,36]  8.485e+03
## reshist_addr1_nanda_parks_a_t_IQR(0.0166,0.0556]:reshist_addr1_coi_he_heat_IQR(15.7,36]    8.482e+03
```

```
## reshist_addr1_nanda_parks_a_t_IQR(0.0556,0.766]:reshist_addr1_coi_he_heat_IQR(15.7,36]     8.441e+03
## reshist_addr1_nanda_parks_a_t_IQR(0.000814,0.0166]:reshist_addr1_coi_he_heat_IQR(36,135]   8.470e+03
## reshist_addr1_nanda_parks_a_t_IQR(0.0166,0.0556]:reshist_addr1_coi_he_heat_IQR(36,135]     8.426e+03
## reshist_addr1_nanda_parks_a_t_IQR(0.0556,0.766]:reshist_addr1_coi_he_heat_IQR(36,135]      8.447e+03
##                                                                                          t value
## (Intercept)                                                                               40.758
## Parent_Ed_lvl                                                                             -1.248
## race_ethnicity.y2                                                                         -0.501
## race_ethnicity.y3                                                                         -1.223
## race_ethnicity.y4                                                                         -1.219
## race_ethnicity.y5                                                                          3.469
## demo_sex_v2.y2                                                                            -1.181
## interview_age                                                                             -1.634
## income_to_needs                                                                           -2.251
## reshist_addr1_years                                                                       -2.712
## reshist_addr1_popdensity_IQR(776,1.61e+03]                                                 1.346
## reshist_addr1_popdensity_IQR(1.61e+03,2.69e+03]                                            1.969
## reshist_addr1_popdensity_IQR(2.69e+03,6.03e+04]                                            0.452
## reshist_addr1_nanda_disadv_fac_IQR(0.0394,0.0687]                                          0.904
## reshist_addr1_nanda_disadv_fac_IQR(0.0687,0.125]                                           2.108
## reshist_addr1_nanda_disadv_fac_IQR(0.125,0.67]                                             2.707
## reshist_addr1_urban_area2                                                                  1.201
## reshist_addr1_urban_area3                                                                  1.375
## reshist_addr1_nanda_parks_a_t_IQR(0.000814,0.0166]                                        -0.352
## reshist_addr1_nanda_parks_a_t_IQR(0.0166,0.0556]                                          -0.030
## reshist_addr1_nanda_parks_a_t_IQR(0.0556,0.766]                                           -0.089
## reshist_addr1_coi_he_heat_IQR(6,15.7]                                                      0.014
## reshist_addr1_coi_he_heat_IQR(15.7,36]                                                     1.010
## reshist_addr1_coi_he_heat_IQR(36,135]                                                      1.930
## reshist_addr1_nanda_parks_a_t_IQR(0.000814,0.0166]:reshist_addr1_coi_he_heat_IQR(6,15.7]  -0.094
## reshist_addr1_nanda_parks_a_t_IQR(0.0166,0.0556]:reshist_addr1_coi_he_heat_IQR(6,15.7]     0.251
## reshist_addr1_nanda_parks_a_t_IQR(0.0556,0.766]:reshist_addr1_coi_he_heat_IQR(6,15.7]      0.441
## reshist_addr1_nanda_parks_a_t_IQR(0.000814,0.0166]:reshist_addr1_coi_he_heat_IQR(15.7,36]  0.532
## reshist_addr1_nanda_parks_a_t_IQR(0.0166,0.0556]:reshist_addr1_coi_he_heat_IQR(15.7,36]   -0.738
## reshist_addr1_nanda_parks_a_t_IQR(0.0556,0.766]:reshist_addr1_coi_he_heat_IQR(15.7,36]    -1.288
## reshist_addr1_nanda_parks_a_t_IQR(0.000814,0.0166]:reshist_addr1_coi_he_heat_IQR(36,135]  -0.806
## reshist_addr1_nanda_parks_a_t_IQR(0.0166,0.0556]:reshist_addr1_coi_he_heat_IQR(36,135]    -1.515
## reshist_addr1_nanda_parks_a_t_IQR(0.0556,0.766]:reshist_addr1_coi_he_heat_IQR(36,135]     -1.478
##                                                                                          Pr(>|t|)
## (Intercept)                                                                              1.09e-14
## Parent_Ed_lvl                                                                            0.212222
```

```
## race_ethnicity.y2                                                                               0.616715
## race_ethnicity.y3                                                                               0.221501
## race_ethnicity.y4                                                                               0.222903
## race_ethnicity.y5                                                                               0.000525
## demo_sex_v2.y2                                                                                  0.237574
## interview_age                                                                                   0.151282
## income_to_needs                                                                                 0.024420
## reshist_addr1_years                                                                             0.006698
## reshist_addr1_popdensity_IQR(776,1.61e+03]                                                      0.178391
## reshist_addr1_popdensity_IQR(1.61e+03,2.69e+03]                                                 0.048992
## reshist_addr1_popdensity_IQR(2.69e+03,6.03e+04]                                                 0.651258
## reshist_addr1_nanda_disadv_fac_IQR(0.0394,0.0687]                                               0.366262
## reshist_addr1_nanda_disadv_fac_IQR(0.0687,0.125]                                                0.035080
## reshist_addr1_nanda_disadv_fac_IQR(0.125,0.67]                                                  0.006811
## reshist_addr1_urban_area2                                                                       0.229629
## reshist_addr1_urban_area3                                                                       0.169293
## reshist_addr1_nanda_parks_a_t_IQR(0.000814,0.0166]                                              0.724774
## reshist_addr1_nanda_parks_a_t_IQR(0.0166,0.0556]                                                0.976446
## reshist_addr1_nanda_parks_a_t_IQR(0.0556,0.766]                                                 0.928826
## reshist_addr1_coi_he_heat_IQR(6,15.7]                                                           0.989189
## reshist_addr1_coi_he_heat_IQR(15.7,36]                                                          0.312304
## reshist_addr1_coi_he_heat_IQR(36,135]                                                           0.053795
## reshist_addr1_nanda_parks_a_t_IQR(0.000814,0.0166]:reshist_addr1_coi_he_heat_IQR(6,15.7]  0.925190
## reshist_addr1_nanda_parks_a_t_IQR(0.0166,0.0556]:reshist_addr1_coi_he_heat_IQR(6,15.7]    0.801727
## reshist_addr1_nanda_parks_a_t_IQR(0.0556,0.766]:reshist_addr1_coi_he_heat_IQR(6,15.7]     0.659070
## reshist_addr1_nanda_parks_a_t_IQR(0.000814,0.0166]:reshist_addr1_coi_he_heat_IQR(15.7,36] 0.594619
## reshist_addr1_nanda_parks_a_t_IQR(0.0166,0.0556]:reshist_addr1_coi_he_heat_IQR(15.7,36]   0.460678
## reshist_addr1_nanda_parks_a_t_IQR(0.0556,0.766]:reshist_addr1_coi_he_heat_IQR(15.7,36]    0.197758
## reshist_addr1_nanda_parks_a_t_IQR(0.000814,0.0166]:reshist_addr1_coi_he_heat_IQR(36,135]  0.420336
## reshist_addr1_nanda_parks_a_t_IQR(0.0166,0.0556]:reshist_addr1_coi_he_heat_IQR(36,135]    0.129895
## reshist_addr1_nanda_parks_a_t_IQR(0.0556,0.766]:reshist_addr1_coi_he_heat_IQR(36,135]     0.139454
##
## (Intercept)                                                                                     ***
## Parent_Ed_lvl
## race_ethnicity.y2
## race_ethnicity.y3
## race_ethnicity.y4
## race_ethnicity.y5                                                                               ***
## demo_sex_v2.y2
## interview_age
## income_to_needs                                                                                 *
```

```
## reshist_addr1_years                                                                             **
## reshist_addr1_popdensity_IQR(776,1.61e+03]
## reshist_addr1_popdensity_IQR(1.61e+03,2.69e+03]                                                 *
## reshist_addr1_popdensity_IQR(2.69e+03,6.03e+04]
## reshist_addr1_nanda_disadv_fac_IQR(0.0394,0.0687]
## reshist_addr1_nanda_disadv_fac_IQR(0.0687,0.125]                                                *
## reshist_addr1_nanda_disadv_fac_IQR(0.125,0.67]                                                  **
## reshist_addr1_urban_area2
## reshist_addr1_urban_area3
## reshist_addr1_nanda_parks_a_t_IQR(0.000814,0.0166]
## reshist_addr1_nanda_parks_a_t_IQR(0.0166,0.0556]
## reshist_addr1_nanda_parks_a_t_IQR(0.0556,0.766]
## reshist_addr1_coi_he_heat_IQR(6,15.7]
## reshist_addr1_coi_he_heat_IQR(15.7,36]
## reshist_addr1_coi_he_heat_IQR(36,135]                                                           .
## reshist_addr1_nanda_parks_a_t_IQR(0.000814,0.0166]:reshist_addr1_coi_he_heat_IQR(6,15.7]
## reshist_addr1_nanda_parks_a_t_IQR(0.0166,0.0556]:reshist_addr1_coi_he_heat_IQR(6,15.7]
## reshist_addr1_nanda_parks_a_t_IQR(0.0556,0.766]:reshist_addr1_coi_he_heat_IQR(6,15.7]
## reshist_addr1_nanda_parks_a_t_IQR(0.000814,0.0166]:reshist_addr1_coi_he_heat_IQR(15.7,36]
## reshist_addr1_nanda_parks_a_t_IQR(0.0166,0.0556]:reshist_addr1_coi_he_heat_IQR(15.7,36]
## reshist_addr1_nanda_parks_a_t_IQR(0.0556,0.766]:reshist_addr1_coi_he_heat_IQR(15.7,36]
## reshist_addr1_nanda_parks_a_t_IQR(0.000814,0.0166]:reshist_addr1_coi_he_heat_IQR(36,135]
## reshist_addr1_nanda_parks_a_t_IQR(0.0166,0.0556]:reshist_addr1_coi_he_heat_IQR(36,135]
## reshist_addr1_nanda_parks_a_t_IQR(0.0556,0.766]:reshist_addr1_coi_he_heat_IQR(36,135]
## ---
## Signif. codes:  0 '***' 0.001 '**' 0.01 '*' 0.05 '.' 0.1 ' ' 1
```

```
##
## Correlation matrix not shown by default, as p = 33 > 12.
## Use print(x, correlation=TRUE)  or
##     vcov(x)        if you need it
```

```
model_performance(Test_IQR)
```

```
## # Indices of model performance
##
## AIC       |      AICc |       BIC | R2 (cond.) | R2 (marg.) |   ICC |  RMSE | Sigma
## ---------------------------------------------------------------------------------
## 2.133e+05 | 2.133e+05 | 2.136e+05 |      0.649 |      0.008 | 0.647 | 4.143 | 4.701
```

```
## Model building- summary and performance comparison
# Unadjusted model (no covariates)
Test_unadjusted <- lmer(sds_p_ss_total ~ reshist_addr1_nanda_parks_a_t * reshist_addr1_coi_he_heat +
                          (1|src_subject_id.x) + (1|eventname.x) + (1|site_id_l),
                        data=SimpleDataset_analytical_z)

# Partially adjusted model (individual-level covariates)
Test_individual <- lmer(sds_p_ss_total ~ Parent_Ed_lvl + race_ethnicity.y + demo_sex_v2.y + interview_age + income_to_needs
+
                          reshist_addr1_nanda_parks_a_t * reshist_addr1_coi_he_heat +
                          (1|src_subject_id.x) + (1|eventname.x) + (1|site_id_l),
                        data=SimpleDataset_analytical_z)

# Partially adjusted model (neighborhood-level covariates)
Test_neighborhood <- lmer(sds_p_ss_total ~ reshist_addr1_years + reshist_addr1_popdensity + reshist_addr1_nanda_disadv_fac +
                            reshist_addr1_urban_area +
                            reshist_addr1_nanda_parks_a_t * reshist_addr1_coi_he_heat +
                            (1|src_subject_id.x) + (1|eventname.x) + (1|site_id_l),
                          data=SimpleDataset_analytical_z)

# Fully adjusted model (current model)
Test_fully_adjusted <- lmer(sds_p_ss_total ~ Parent_Ed_lvl + race_ethnicity.y + demo_sex_v2.y + interview_age + income_to_ne
eds +
                              reshist_addr1_years + reshist_addr1_popdensity + reshist_addr1_nanda_disadv_fac + reshist_addr
1_urban_area +
                              reshist_addr1_nanda_parks_a_t * reshist_addr1_coi_he_heat +
                              (1|src_subject_id.x) + (1|eventname.x) + (1|site_id_l),
                            data=SimpleDataset_analytical_z)

# Model summaries and performance
summary(Test_unadjusted)
```

```
## Linear mixed model fit by REML. t-tests use Satterthwaite's method [
## lmerModLmerTest]
## Formula:
## sds_p_ss_total ~ reshist_addr1_nanda_parks_a_t * reshist_addr1_coi_he_heat +
##     (1 | src_subject_id.x) + (1 | eventname.x) + (1 | site_id_l)
##    Data: SimpleDataset_analytical_z
##
## REML criterion at convergence: 79066.8
##
## Scaled residuals:
##     Min      1Q  Median      3Q     Max
## -6.2590 -0.4979 -0.1074  0.4020 14.1948
##
## Random effects:
##  Groups           Name        Variance Std.Dev.
##  src_subject_id.x (Intercept) 0.63489  0.79680
##  site_id_l        (Intercept) 0.01336  0.11557
##  eventname.x      (Intercept) 0.00238  0.04879
##  Residual                     0.35893  0.59911
## Number of obs: 33816, groups:
## src_subject_id.x, 8797; site_id_l, 22; eventname.x, 5
##
## Fixed effects:
##                                                          Estimate Std. Error
## (Intercept)                                            -8.273e-03  3.456e-02
## reshist_addr1_nanda_parks_a_t                          -2.199e-02  9.570e-03
## reshist_addr1_coi_he_heat                               4.967e-02  1.719e-02
## reshist_addr1_nanda_parks_a_t:reshist_addr1_coi_he_heat -1.987e-02  1.056e-02
##                                                                df t value
## (Intercept)                                             1.677e+01  -0.239
## reshist_addr1_nanda_parks_a_t                           8.805e+03  -2.298
## reshist_addr1_coi_he_heat                               2.301e+02   2.889
## reshist_addr1_nanda_parks_a_t:reshist_addr1_coi_he_heat  8.984e+03  -1.882
##                                                        Pr(>|t|)
## (Intercept)                                             0.81368
## reshist_addr1_nanda_parks_a_t                           0.02160 *
## reshist_addr1_coi_he_heat                               0.00423 **
## reshist_addr1_nanda_parks_a_t:reshist_addr1_coi_he_heat  0.05990 .
## ---
## Signif. codes:  0 '***' 0.001 '**' 0.01 '*' 0.05 '.' 0.1 ' ' 1
##
```

```
## Correlation of Fixed Effects:
##              (Intr) rs_1____ rs_1___
## rshst_1____ 0.008
## rshst_d1___ 0.014  0.070
## r_1____:_1_ 0.025  0.228    0.052
```

```
summary(Test_individual)
```

```
 # Linear mixed model fit by REML. t-tests use Satterthwaite's method [
## lmerModLmerTest]
## Formula: sds_p_ss_total ~ Parent_Ed_lvl + race_ethnicity.y + demo_sex_v2.y +
##     interview_age + income_to_needs + reshist_addr1_nanda_parks_a_t *
##     reshist_addr1_coi_he_heat + (1 | src_subject_id.x) + (1 |
##     eventname.x) + (1 | site_id_l)
##    Data: SimpleDataset_analytical_z
##
## REML criterion at convergence: 79054.4
##
## Scaled residuals:
##     Min      1Q  Median      3Q     Max
## -6.2761 -0.4966 -0.1061  0.4017 14.1875
##
## Random effects:
##  Groups           Name        Variance  Std.Dev.
##  src_subject_id.x (Intercept) 0.6317998 0.79486
##  site_id_l        (Intercept) 0.0120986 0.10999
##  eventname.x      (Intercept) 0.0008666 0.02944
##  Residual                     0.3590407 0.59920
## Number of obs: 33808, groups:
## src_subject_id.x, 8794; site_id_l, 22; eventname.x, 5
##
## Fixed effects:
##                                                          Estimate Std. Error
## (Intercept)                                            -3.588e-03  3.179e-02
## Parent_Ed_lvl                                          -8.560e-03  5.686e-03
## race_ethnicity.y2                                       3.109e-02  3.123e-02
## race_ethnicity.y3                                      -2.769e-02  2.868e-02
## race_ethnicity.y4                                      -8.454e-02  6.290e-02
## race_ethnicity.y5                                       1.251e-01  3.153e-02
## demo_sex_v2.y2                                         -2.095e-02  1.837e-02
## interview_age                                          -2.278e-02  1.122e-02
## income_to_needs                                        -2.641e-02  7.917e-03
## reshist_addr1_nanda_parks_a_t                          -2.139e-02  9.572e-03
## reshist_addr1_coi_he_heat                               4.426e-02  1.699e-02
## reshist_addr1_nanda_parks_a_t:reshist_addr1_coi_he_heat -2.086e-02  1.057e-02
##                                                                df t value
## (Intercept)                                             2.909e+01  -0.113
## Parent_Ed_lvl                                           3.170e+04  -1.505
## race_ethnicity.y2                                       9.107e+03   0.996
```

```
## race_ethnicity.y3                                      5.539e+03  -0.965
## race_ethnicity.y4                                      8.370e+03  -1.344
## race_ethnicity.y5                                      8.640e+03   3.967
## demo_sex_v2.y2                                         8.619e+03  -1.141
## interview_age                                          6.593e+00  -2.030
## income_to_needs                                        2.651e+04  -3.336
## reshist_addr1_nanda_parks_a_t                          8.787e+03  -2.234
## reshist_addr1_coi_he_heat                              2.076e+02   2.605
## reshist_addr1_nanda_parks_a_t:reshist_addr1_coi_he_heat  8.956e+03  -1.974
##                                                       Pr(>|t|)
## (Intercept)                                           0.910905
## Parent_Ed_lvl                                         0.132231
## race_ethnicity.y2                                     0.319461
## race_ethnicity.y3                                     0.334395
## race_ethnicity.y4                                     0.178970
## race_ethnicity.y5                                     7.34e-05 ***
## demo_sex_v2.y2                                        0.254011
## interview_age                                         0.084450 .
## income_to_needs                                       0.000852 ***
## reshist_addr1_nanda_parks_a_t                         0.025480 *
## reshist_addr1_coi_he_heat                             0.009862 **
## reshist_addr1_nanda_parks_a_t:reshist_addr1_coi_he_heat 0.048379 *
## ---
## Signif. codes:  0 '***' 0.001 '**' 0.01 '*' 0.05 '.' 0.1 ' ' 1
##
## Correlation of Fixed Effects:
##             (Intr) Prn_E_ rc_t.2 rc_t.3 rc_t.4 rc_t.5 d__2.2 intrv_ incm__
## Prnt_Ed_lvl -0.023
## rc_thncty.2 -0.208  0.074
## rc_thncty.3 -0.245  0.112  0.275
## rc_thncty.4 -0.098 -0.017  0.085  0.137
## rc_thncty.5 -0.182  0.015  0.211  0.240  0.104
## dm_sx_v2.y2 -0.273  0.003 -0.015 -0.001 -0.011 -0.001
## interview_g -0.069 -0.046 -0.016 -0.007 -0.006 -0.007  0.015
## incom_t_nds -0.069 -0.134  0.240  0.200  0.003  0.080 -0.010 -0.044
## rshst_1____  0.005 -0.020 -0.024  0.037  0.011 -0.005  0.001 -0.003 -0.033
## rshst_d1___  0.025 -0.006 -0.042 -0.001 -0.012 -0.021 -0.010  0.001  0.048
## r_1____:_1_  0.046  0.009 -0.059 -0.046 -0.024 -0.017  0.003 -0.008  0.004
##             rs_1____ rs_1___
## Prnt_Ed_lvl
## rc_thncty.2
```

```
## rc_thncty.3
## rc_thncty.4
## rc_thncty.5
## dm_sx_v2.y2
## interview_g
## incom_t_nds
## rshst_1____
## rshst_d1___  0.070
## r_1____:_1_  0.225    0.057
```

```
summary(Test_neighborhood)
```

```
## Linear mixed model fit by REML. t-tests use Satterthwaite's method [
## lmerModLmerTest]
## Formula: sds_p_ss_total ~ reshist_addr1_years + reshist_addr1_popdensity +
##     reshist_addr1_nanda_disadv_fac + reshist_addr1_urban_area +
##     reshist_addr1_nanda_parks_a_t * reshist_addr1_coi_he_heat +
##     (1 | src_subject_id.x) + (1 | eventname.x) + (1 | site_id_l)
##    Data: SimpleDataset_analytical_z
##
## REML criterion at convergence: 76978.6
##
## Scaled residuals:
##     Min      1Q  Median      3Q     Max
## -6.3237 -0.4980 -0.1074  0.4033 14.2674
##
## Random effects:
##  Groups           Name        Variance Std.Dev.
##  src_subject_id.x (Intercept) 0.635989 0.79749
##  site_id_l        (Intercept) 0.013046 0.11422
##  eventname.x      (Intercept) 0.002287 0.04783
##  Residual                     0.355319 0.59609
## Number of obs: 33018, groups:
## src_subject_id.x, 8583; site_id_l, 22; eventname.x, 5
##
## Fixed effects:
##                                                          Estimate Std. Error
## (Intercept)                                             2.226e-02  3.749e-02
## reshist_addr1_years                                    -6.858e-03  2.544e-03
## reshist_addr1_popdensity                                1.290e-02  1.005e-02
## reshist_addr1_nanda_disadv_fac                          3.899e-02  1.017e-02
## reshist_addr1_urban_area2                               6.084e-02  5.300e-02
## reshist_addr1_urban_area3                               4.026e-02  3.507e-02
## reshist_addr1_nanda_parks_a_t                          -1.950e-02  9.687e-03
## reshist_addr1_coi_he_heat                               5.087e-02  1.740e-02
## reshist_addr1_nanda_parks_a_t:reshist_addr1_coi_he_heat -2.298e-02  1.073e-02
##                                                                df t value
## (Intercept)                                             2.462e+01   0.594
## reshist_addr1_years                                     8.455e+03  -2.696
## reshist_addr1_popdensity                                6.555e+03   1.284
## reshist_addr1_nanda_disadv_fac                          6.578e+03   3.834
## reshist_addr1_urban_area2                               8.311e+03   1.148
## reshist_addr1_urban_area3                               7.962e+03   1.148
```

```
## reshist_addr1_nanda_parks_a_t                              8.582e+03  -2.013
## reshist_addr1_coi_he_heat                                  2.234e+02   2.925
## reshist_addr1_nanda_parks_a_t:reshist_addr1_coi_he_heat  8.769e+03  -2.142
##                                                           Pr(>|t|)
## (Intercept)                                               0.558067
## reshist_addr1_years                                       0.007027 **
## reshist_addr1_popdensity                                  0.199310
## reshist_addr1_nanda_disadv_fac                            0.000127 ***
## reshist_addr1_urban_area2                                 0.250960
## reshist_addr1_urban_area3                                 0.250948
## reshist_addr1_nanda_parks_a_t                             0.044109 *
## reshist_addr1_coi_he_heat                                 0.003804 **
## reshist_addr1_nanda_parks_a_t:reshist_addr1_coi_he_heat 0.032242 *
## ---
## Signif. codes:  0 '***' 0.001 '**' 0.01 '*' 0.05 '.' 0.1 ' ' 1
##
## Correlation of Fixed Effects:
##                 (Intr) rshst_ddr1_y rshst_ddr1_p rshst_ddr1_n__ r_1__2 r_1__3
## rshst_ddr1_y   -0.399
## rshst_ddr1_p   -0.035 -0.002
## rshst_ddr1_n__ -0.093  0.151       -0.192
## rshst_d1__2    -0.062  0.008        0.062        0.022
## rshst_d1__3    -0.107  0.021        0.149        0.115          0.133
## rshst_1____     0.000 -0.004        0.048        0.007          0.032  0.073
## rshst_ddr1_c__  0.002  0.031        0.073       -0.054          0.012 -0.021
## r_1____:_1_     0.021 -0.009       -0.012       -0.062          0.044  0.065
##                 rs_1____ rshst_ddr1_c__
## rshst_ddr1_y
## rshst_ddr1_p
## rshst_ddr1_n__
## rshst_d1__2
## rshst_d1__3
## rshst_1____
## rshst_ddr1_c__  0.071
## r_1____:_1_     0.233    0.052
```

```
summary(Test_fully_adjusted)
```

```
## Linear mixed model fit by REML. t-tests use Satterthwaite's method [
## lmerModLmerTest]
## Formula: sds_p_ss_total ~ Parent_Ed_lvl + race_ethnicity.y + demo_sex_v2.y +
##     interview_age + income_to_needs + reshist_addr1_years + reshist_addr1_popdensity +
##     reshist_addr1_nanda_disadv_fac + reshist_addr1_urban_area +
##     reshist_addr1_nanda_parks_a_t * reshist_addr1_coi_he_heat +
##     (1 | src_subject_id.x) + (1 | eventname.x) + (1 | site_id_l)
##    Data: SimpleDataset_analytical_z
##
## REML criterion at convergence: 76980
##
## Scaled residuals:
##     Min      1Q  Median      3Q     Max
## -6.3212 -0.4979 -0.1062  0.4031 14.2600
##
## Random effects:
##  Groups           Name        Variance Std.Dev.
##  src_subject_id.x (Intercept) 0.63432  0.79644
##  site_id_l        (Intercept) 0.01227  0.11075
##  eventname.x      (Intercept) 0.00103  0.03209
##  Residual                     0.35537  0.59613
## Number of obs: 33010, groups:
## src_subject_id.x, 8580; site_id_l, 22; eventname.x, 5
##
## Fixed effects:
##                                                          Estimate Std. Error
## (Intercept)                                             3.918e-02  3.685e-02
## Parent_Ed_lvl                                          -6.109e-03  5.778e-03
## race_ethnicity.y2                                      -4.358e-02  3.735e-02
## race_ethnicity.y3                                      -4.132e-02  2.979e-02
## race_ethnicity.y4                                      -7.268e-02  6.341e-02
## race_ethnicity.y5                                       1.075e-01  3.238e-02
## demo_sex_v2.y2                                         -2.058e-02  1.861e-02
## interview_age                                          -1.905e-02  1.185e-02
## income_to_needs                                        -1.885e-02  8.200e-03
## reshist_addr1_years                                    -6.439e-03  2.578e-03
## reshist_addr1_popdensity                                1.301e-02  1.011e-02
## reshist_addr1_nanda_disadv_fac                          3.721e-02  1.236e-02
## reshist_addr1_urban_area2                               5.361e-02  5.311e-02
## reshist_addr1_urban_area3                               3.646e-02  3.509e-02
## reshist_addr1_nanda_parks_a_t                          -1.915e-02  9.697e-03
```

```
## reshist_addr1_coi_he_heat                                4.891e-02  1.727e-02
## reshist_addr1_nanda_parks_a_t:reshist_addr1_coi_he_heat -2.210e-02  1.073e-02
##                                                                 df t value
## (Intercept)                                              4.148e+01   1.063
## Parent_Ed_lvl                                            3.090e+04  -1.057
## race_ethnicity.y2                                        8.776e+03  -1.167
## race_ethnicity.y3                                        5.987e+03  -1.387
## race_ethnicity.y4                                        8.182e+03  -1.146
## race_ethnicity.y5                                        8.384e+03   3.321
## demo_sex_v2.y2                                           8.405e+03  -1.106
## interview_age                                            6.224e+00  -1.608
## income_to_needs                                          2.765e+04  -2.299
## reshist_addr1_years                                      8.484e+03  -2.498
## reshist_addr1_popdensity                                 6.974e+03   1.287
## reshist_addr1_nanda_disadv_fac                           8.287e+03   3.009
## reshist_addr1_urban_area2                                8.314e+03   1.009
## reshist_addr1_urban_area3                                7.934e+03   1.039
## reshist_addr1_nanda_parks_a_t                            8.567e+03  -1.975
## reshist_addr1_coi_he_heat                                2.095e+02   2.832
## reshist_addr1_nanda_parks_a_t:reshist_addr1_coi_he_heat  8.744e+03  -2.060
##                                                         Pr(>|t|)
## (Intercept)                                              0.29380
## Parent_Ed_lvl                                            0.29040
## race_ethnicity.y2                                        0.24334
## race_ethnicity.y3                                        0.16556
## race_ethnicity.y4                                        0.25175
## race_ethnicity.y5                                        0.00090 ***
## demo_sex_v2.y2                                           0.26891
## interview_age                                            0.15715
## income_to_needs                                          0.02152 *
## reshist_addr1_years                                      0.01252 *
## reshist_addr1_popdensity                                 0.19818
## reshist_addr1_nanda_disadv_fac                           0.00263 **
## reshist_addr1_urban_area2                                0.31282
## reshist_addr1_urban_area3                                0.29881
## reshist_addr1_nanda_parks_a_t                            0.04831 *
## reshist_addr1_coi_he_heat                                0.00508 **
## reshist_addr1_nanda_parks_a_t:reshist_addr1_coi_he_heat  0.03941 *
## ---
## Signif. codes:  0 '***' 0.001 '**' 0.01 '*' 0.05 '.' 0.1 ' ' 1
```

```
##
## Correlation matrix not shown by default, as p = 17 > 12.
## Use print(x, correlation=TRUE)  or
##     vcov(x)        if you need it
```

```
# Model performance comparisons
model_performance(Test_unadjusted)
```

```
## # Indices of model performance
##
## AIC       |      AICc |       BIC | R2 (cond.) | R2 (marg.) |   ICC |  RMSE | Sigma
## ------------------------------------------------------------------------------------
## 79082.764 | 79082.768 | 79150.193 |      0.646 |      0.003 | 0.644 | 0.528 | 0.599
```

```
model_performance(Test_individual)
```

```
## # Indices of model performance
##
## AIC       |      AICc |       BIC | R2 (cond.) | R2 (marg.) |   ICC |  RMSE | Sigma
## ------------------------------------------------------------------------------------
## 79086.393 | 79086.409 | 79221.248 |      0.645 |      0.008 | 0.642 | 0.528 | 0.599
```

```
model_performance(Test_neighborhood)
```

```
## # Indices of model performance
##
## AIC       |      AICc |       BIC | R2 (cond.) | R2 (marg.) |   ICC |  RMSE | Sigma
## ------------------------------------------------------------------------------------
## 77004.596 | 77004.607 | 77113.859 |      0.649 |      0.007 | 0.647 | 0.525 | 0.596
```

```
model_performance(Test_fully_adjusted)
```

```
# # Indices of model performance
#
# AIC       |      AICc |       BIC | R2 (cond.) | R2 (marg.) |   ICC |  RMSE | Sigma
# -----------------------------------------------------------------------------------
# 77022.028 | 77022.056 | 77198.524 |      0.649 |      0.010 | 0.646 | 0.525 | 0.596
```
